# Alpinetin Nanoparticles Alleviate Optic Nerve Injury Induced by Acute Glaucoma via LRP1‐PPARγ Mediated Regulation of Microglial Lipid Metabolism

**DOI:** 10.1002/advs.202513270

**Published:** 2025-11-12

**Authors:** Miao Wei, Yujia Huo, Jingchang Yuan, Xiao Fan, Xiaochen Wang, Sisi Tan, Xi Gao, Ruotong Ouyang, Hong Li

**Affiliations:** ^1^ Department of Ophthalmology The First Affiliated Hospital of Chongqing Medical University Chongqing P. R. China; ^2^ Chongqing Key Laboratory for the Prevention and Treatment of Major Blinding Eye Diseases Chongqing Eye Institute Chongqing Branch of National Clinical Research Center for Ocular Diseases Chongqing P. R. China

**Keywords:** AlpNPs, glaucoma, lipid metabolism, LRP1‐PPARγ, microglia polarization

## Abstract

Glaucoma is a leading cause of irreversible blindness, characterized by progressive retinal ganglion cells (RGCs) loss. Increasing evidence links microglial activation and lipid metabolism dysregulation to neurodegeneration. However, the role of microglial lipid metabolic reprogramming in disease pathogenesis remains unclear. This study finds that microglia in an acute ocular hypertension (AOH) model exhibit abnormal lipid droplet accumulation, downregulation of low‐density lipoprotein receptor‐related protein 1 (LRP1), and a shift toward a pro‐inflammatory M1 phenotype. Importantly, serum samples from glaucoma patients reveal significantly reduced LRP1 levels compared to controls. To restore lipid homeostasis, this study develops alpinetin‐loaded PLGA nanoparticles (AlpNPs), which demonstrate efficient microglial uptake and sustained release. AlpNPs reduced intracellular lipid accumulation, promoted M2 polarization, and suppressed microglial proliferation and migration. Mechanistically, AlpNPs directly bind to LRP1 and enhance its interaction with PPARγ, thereby activating the downstream LXRα‐ABCA1 pathway, which is pivotal for cholesterol efflux and anti‐inflammatory responses. Knockdown of LRP1 abolished the protective effects of AlpNPs, confirming its essential role in mediating metabolic reprogramming. In vivo, intravitreal injection of AlpNPs significantly attenuates retinal inflammation and preserves RGCs in AOH. These findings identify microglial lipid metabolic dysfunction as a key driver in glaucoma and highlight LRP1‐targeted nanotherapy as a promising strategy for neuroprotection.

## Graphical abstract

Microglial lipid metabolic dysfunction drives neurodegeneration in glaucoma. We found loss of LRP1 causes lipid accumulation and inflammation. We developed alpinetin‐loaded nanoparticles (AlpNPs) that bind LRP1, activate the PPARγ‐LXRα‐ABCA1 pathway to restore lipid homeostasis, promote an anti‐inflammatory phenotype, and protect retinal ganglion cells, offering a novel nanotherapy strategy.

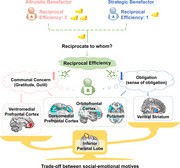

## 1 Introduction

Glaucoma is a neurodegenerative disease and the leading cause of irreversible blindness worldwide, projected to affect over 112 million individuals by 2040.^[^ ^1^ ^]^ Characterized by the progressive loss of retinal ganglion cells (RGCs) and optic nerve axons, glaucoma leads to gradual visual field defects and ultimately irreversible vision loss.^[^ ^2^ ^]^ Although elevated intraocular pressure (IOP) remains the most established risk factor, clinical observations have revealed that a significant proportion of patients continue to experience visual deterioration despite well‐controlled IOP, indicating the involvement of IOP‐independent mechanisms in glaucomatous neurodegeneration.^[^ ^3^ ^]^ Thus, a deeper understanding of the underlying molecular and cellular processes beyond IOP elevation is urgently needed to develop novel neuroprotective therapies.

Among various contributing factors, neuroinflammation has emerged as a key pathological hallmark of glaucomatous injury.^[^ ^4^ ^]^ Retinal microglia, the resident innate immune cells of the central nervous system, play a dual role in maintaining retinal homeostasis and initiating immune responses to injury or stress.^[^ ^5^, ^6^ ^]^ Under physiological conditions, microglia remain in a surveillant state, actively monitoring the microenvironment through their dynamic processes. However, in response to glaucomatous stimuli such as mechanical stress, oxidative damage, or metabolic disruption, microglia rapidly transform into reactive phenotypes, often categorized into classical M1‐like (pro‐inflammatory) and alternative M2‐like (anti‐inflammatory and reparative) states.^[^ ^7^ ^]^ The balance between these polarization states profoundly influences neuronal fate: M1 microglia release pro‐inflammatory cytokines such as TNF‐α and IL‐1β, exacerbating neuronal injury, while M2 microglia secrete anti‐inflammatory mediators like IL‐10 and ARG1, promoting tissue repair and neuroprotection.^[^ ^8^ ^]^

While the role of microglial activation in glaucoma has been extensively studied, the upstream mechanisms that dictate microglial phenotypic transitions remain incompletely defined. Recent advances suggest that cellular metabolism, particularly lipid metabolism, is a crucial determinant of immune cell function and fate.^[^ ^9^ ^]^ Microglia require tightly regulated lipid uptake, storage, and degradation processes to maintain their immunological homeostasis and phagocytic capacity. Aberrant lipid metabolism, especially lipid droplet (LD) accumulation, has been associated with impaired microglial function and increased neuroinflammation in neurodegenerative diseases such as Alzheimer's disease (AD) and Parkinson's disease.^[^ ^10^, ^11^, ^12^ ^]^ However, whether similar lipid metabolic dysfunctions occur in retinal microglia during glaucoma, and how these perturbations influence their polarization and neurotoxic potential, remain poorly understood.

In this context, low‐density lipoprotein receptor‐related protein 1 (LRP1) has gained increasing recognition as a critical regulator of lipid homeostasis and inflammation. LRP1 is a multifunctional endocytic receptor that facilitates lipid uptake and modulates intracellular signaling pathways.^[^ ^13^ ^]^ Notably, LRP1 interacts with peroxisome proliferator‐activated receptor gamma (PPARγ), a master regulator of lipid metabolism and anti‐inflammatory gene expression. Activation of PPARγ initiates key downstream pathways, such as the LXRα‐ABCA1 axis, which promotes cholesterol reverse transport.^[^ ^14^ ^]^ Previous studies have demonstrated that LRP1 promotes cholesterol efflux, reduces LD accumulation, and drives M2‐like polarization in macrophages and central nervous system microglia.^[^ ^15^, ^16^, ^17^ ^]^ Despite its neuroprotective roles in other brain pathologies, the relevance of LRP1‐PPARγ signaling in retinal microglia under glaucomatous stress has not been elucidated.

Here, we present clinical and experimental evidence supporting the hypothesis that LRP1 downregulation and associated lipid metabolic dysfunction in microglia contribute to glaucomatous RGC degeneration. Serum samples from patients with primary open‐angle glaucoma (POAG) and primary angle‐closure glaucoma (PACG) revealed significantly reduced levels of LRP1 compared to age‐related cataract (ARC) controls, indicating a potential systemic signature of lipid metabolic disruption in glaucoma. In an acute ocular hypertension (AOH) model, we observed marked LD accumulation and a shift toward the M1‐like phenotype in retinal microglia, along with decreased LRP1 expression. These findings suggest that impaired lipid clearance via LRP1 downregulation may initiate or exacerbate microglial‐mediated neuroinflammation in glaucoma.

To target this mechanism, we sought a safe and effective therapeutic candidate capable of simultaneously modulating microglial inflammation and lipid metabolism. We focused on the natural flavonoid compound Alpinetin (Alp, PubChem CID: 154 279, Figure 2A).^[^ ^18^ ^]^ Alp has demonstrated diverse pharmacologic activities in preclinical studies, including potent anti‐neuroinflammatory, antioxidant, and immunometabolic regulatory effects.^[^ ^19^, ^20^, ^21^ ^]^ For instance, in models of spinal cord injury and atherosclerosis, it has been shown to effectively suppress the overactivation of microglia/macrophages, promote their polarization toward a protective M2 phenotype, and mitigate neuronal damage and lipid accumulation.^[^ ^19^, ^20^, ^22^, ^23^ ^]^ Owing to its favorable anti‐apoptotic and anti‐inflammatory properties, Alp has also been utilized in various organ ischemia‐reperfusion diseases.^[^ ^23^, ^24^ ^]^ Despite this promising profile, its application in ophthalmic diseases, particularly glaucoma, remains unexplored. Furthermore, its inherent poor aqueous solubility and low bioavailability present significant challenges for clinical translation. In recent years, nanomedicine, which integrates nanotechnology with pharmaceutical and biomedical sciences, has gained considerable attention.^[^ ^19^ ^]^ Poly (lactic‐co‐glycolic acid) (PLGA) nanoparticles are widely used polymeric carriers capable of encapsulating hydrophobic drugs within an organic core surrounded by a hydrophilic shell. This shell can potentially carry a second hydrophilic active ingredient. PLGA nanoparticles offer high drug encapsulation efficiency, sustained‐release properties, and controlled kinetic parameters.^[^ ^25^ ^]^ Encapsulation of alpinetin‐loaded PLGA nanoparticles (AlpNPs) enhances its bioavailability and pharmacokinetic profile, thereby enabling controlled release, minimizing the required drug dosage, and reducing potential systemic toxicity.

In this study, we demonstrate that AlpNPs selectively bind to microglial LRP1, restore LRP1‐PPARγ signaling, and subsequently activate the downstream LXRα‐ABCA1 pathway, thereby suppressing lipid accumulation, and promoting anti‐inflammatory M2‐like polarization. These effects ultimately lead to a reduction in RGCs loss in the AOH model. Furthermore, targeted knockdown of LRP1 abolished the therapeutic benefits of AlpNPs, confirming the essential role of the LRP1‐PPARγ axis in mediating microglial immunometabolic reprogramming and retinal neuroprotection. Our findings unveil a previously underappreciated role of microglial lipid metabolism in glaucoma, delineating a complete LRP1‐PPARγ‐LXRα‐ABCA1 signaling pathway, and establish LRP1 as a novel therapeutic target. By integrating nanomedicine with mechanistic immunometabolism, this work offers a promising strategy to counteract vision loss in glaucoma and potentially other neurodegenerative diseases involving microglial dysfunction.

## 2 Results

### 2.1 Abnormal Lipid Accumulation and Inflammatory Activation of Microglia in AOH

**Figure 1:**
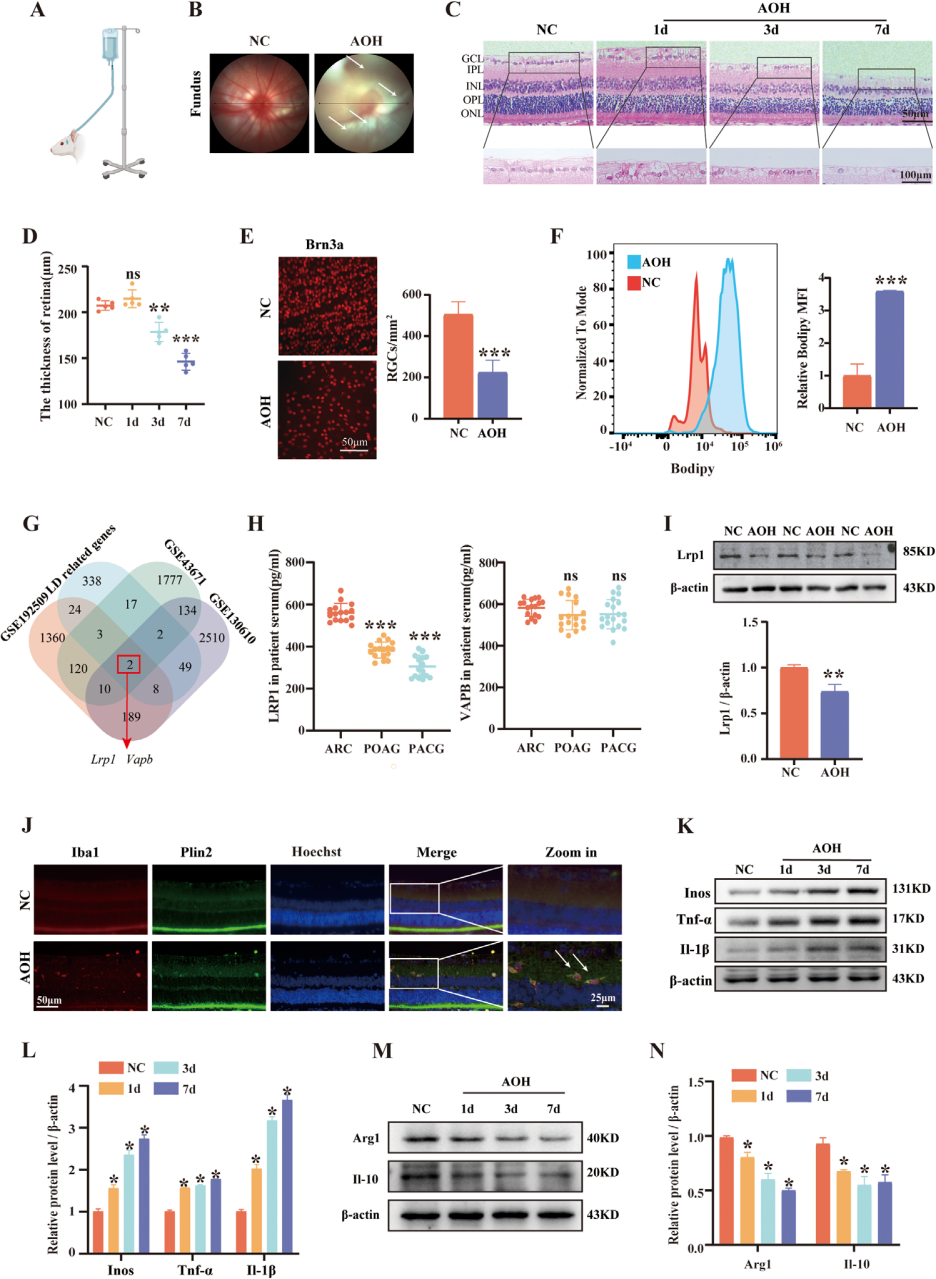
AOH Induces Lipid Accumulation, Inflammatory Activation in Retinal Microglia, and RGCs Loss. A) Schematic illustration of the AOH model. B) Representative fundus photographs from the NC and AOH groups. The images from the AOH group reveal vascular attenuation (indicated by white arrows) and suggestive decreased perfusion. C,D) Representative H&E‐stained retinal sections showing retinal thinning in the AOH group compared to controls. Scale bar: 50 µm (*n* = 5). E) Quantification of RGCs numbers in each group (*n* = 5). F) Flow cytometry analysis of mean fluorescence intensity of LD in retinal cells. Scale bar: 50 µm (*n* = 3). G) Venn diagram of LD‐associated genes identified from GEO datasets. H) ELISA quantification of LRP1 and VAPB protein levels in the serum of glaucoma patients versus healthy controls. I) Representative immunoblot and quantitative analysis of Lrp1 protein levels in retinal whole lysates. J) Representative immunofluorescence images showing colocalization of Plin2 with Iba1+ microglia in the retina. Scale bar: 50 µm(*n* = 4). K–N) Western blotting analysis of inflammatory and anti‐inflammatory cytokines in the retina. Protein levels were quantified using ImageJ software and normalized to β‐actin. Data are presented as mean ± SD from three independent experiments. Statistical significance was determined using one‐way ANOVA. ^*^ *p* <0.05; ^**^ *p* <0.01; ^***^ *p* <0.001; ns, not significant.

To investigate the pathogenesis of glaucoma, we first established an AOH model, a widely accepted and classical animal model for glaucoma research (**Figure** **1**A). As shown in Figure S1A (Supporting Information), the highest IOP in the model group was approximately 98 mmHg during the ischemic period, and it returned to normal during the reperfusion period, which was consistent with the pathological process of acute ocular hypertension. Furthermore, fundus examination revealed pallor and attenuated blood vessels, indicative of ischemia, exclusively in the AOH group (Figure 1B). Hematoxylin and Eosin (H&E) staining revealed retinal neuroepithelial edema and structural disorganization as early as day 1 post‐induction. As the duration of elevated intraocular pressure increased, retinal thickness was significantly reduced, accompanied by a marked decrease in ganglion cell layer (GCL) cell count and RGCs numbers (Figure 1C–E). Previous studies have demonstrated that the accumulation of LD in microglia is closely associated with alterations in their inflammatory phenotype.^[^ ^26^ ^]^ To determine whether lipid metabolic abnormalities occur in the AOH model, we measured the mean fluorescence intensity of LD in retinal cells using flow cytometry.^[^ ^27^ ^]^ Assessment of lipid accumulation revealed a significant increase in LD in the retinas of AOH group, as demonstrated by both flow cytometric quantification (Figure 1F) and direct visualization on retinal flat mounts (Figure S1B, Supporting Information).

To identify key LD metabolism‐related genes involved in glaucoma, we analyzed three publicly available glaucoma transcriptomic datasets from the GEO database: GSE130610, GSE192509, and GSE43671. differentially expressed genes (DEGs) were identified using the “limma” R package with thresholds set at |logFC| > 0.5 and *p* <0.05. DEGs were then intersected with a predefined set of LD‐related genes, including lipid metabolism and cholesterol‐associated genes. This analysis yielded two key candidate genes associated with lipid metabolic alterations in glaucoma: *Lrp1* and *Vapb* (Figure 1G). To further investigate whether glaucoma affects the peripheral expression levels of these genes, we collected serum samples from patients with POAG with ARC, PACG with ARC, as well as ARC‐only controls. Protein concentrations of LRP1 and VAPB were measured by the enzyme‐linked immunosorbent assay (ELISA). Compared to the control group, serum LRP1 levels were significantly decreased in glaucoma patients, while VAPB levels showed no significant difference (Figure 1H; Table S1, Supporting Information). Consistently, a reduction in Lrp1 protein expression was also observed in the retinas of AOH group (Figure 1I). To further verify whether microglial lipid metabolism was impaired, we examined the expression of the lipid droplet‐associated protein Perilipin‐2 (PLIN2). Immunofluorescence staining revealed a pronounced colocalization of Plin2 with the microglial marker Iba1 in AOH retinas, indicating enhanced LD accumulation within microglia (Figure 1J). Given the pivotal role of microglial polarization in the pathogenesis of glaucoma, we next assessed the expression of inflammatory cytokines in retinal tissue by western blotting. Pro‐inflammatory markers (Tnf‐α, Il‐1β, Inos) were significantly upregulated in the AOH group, whereas anti‐inflammatory markers (Il‐10, Arg1) were markedly downregulated (Figure 1K–N). Collectively, these findings suggest that microglia in the AOH model exhibit both aberrant LD accumulation and dysregulated inflammatory polarization, which may contribute critically to RGCs damage in glaucomatous pathology.

### 2.2 Synthesis and Characterization of AlpNPs

**Figure 2:**
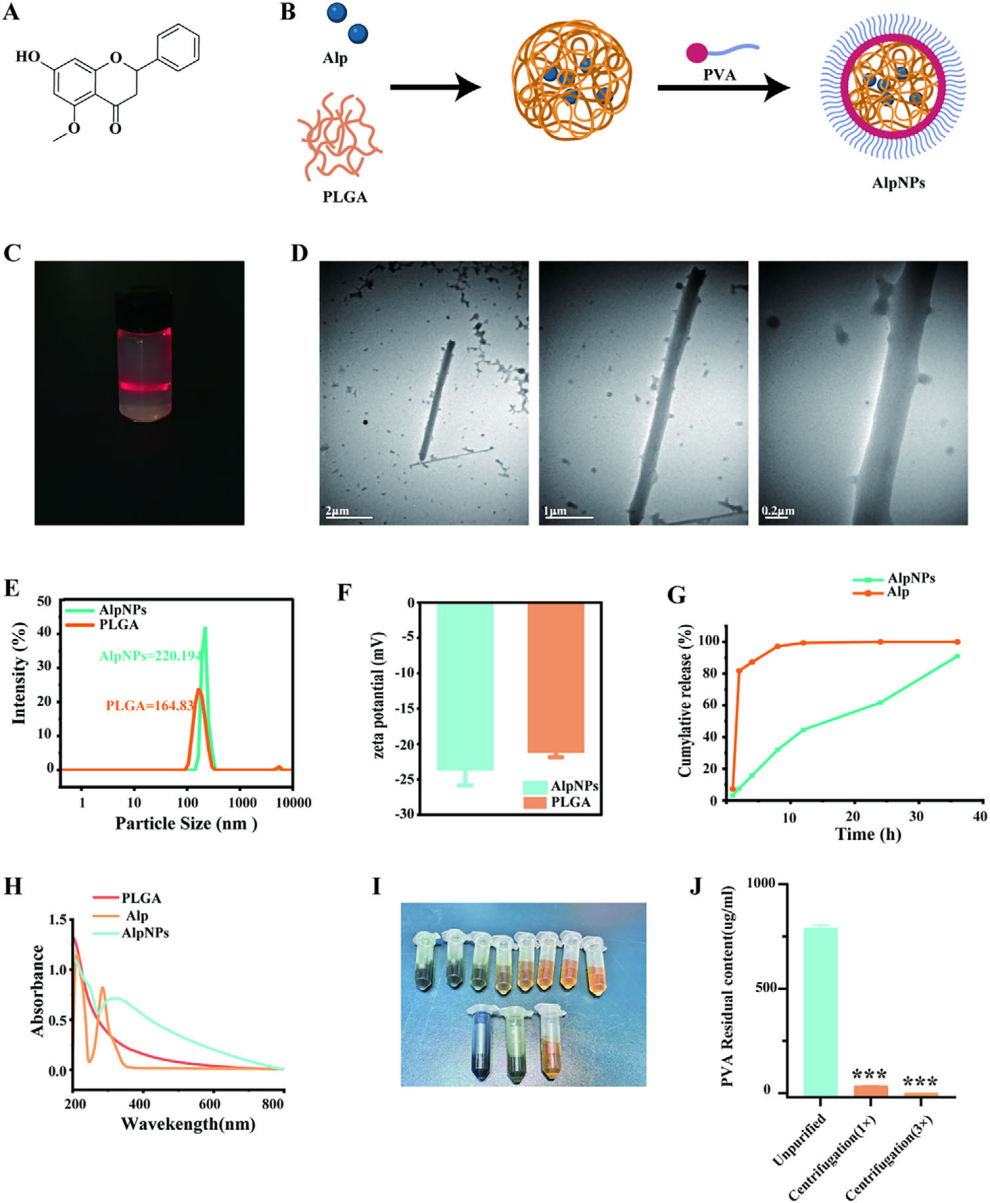
Synthesis and Characterization of AlpNPs. A) Chemical structure of Alp. B) Schematic illustration of the preparation process of AlpNPs. C) Photograph of the colloidal suspension of AlpNPs. D) Representative TEM images of AlpNPs at different magnifications, showing their spherical and monodisperse morphology. Scale bars: 2µm (left),1µm (middle), 0.2µm (right). E,F) DLS analysis showing the hydrodynamic diameter and zeta potential of AlpNPs. G) In vitro release profile of Alp from AlpNPs. H) UV–vis absorption spectra of blank PLGA nanoparticles, free Alp, and AlpNPs. The spectrum of AlpNPs confirms the successful loading of Alp into the nanoparticles. I) Assessment of PVA removal. (Top) Standard curve samples for PVA quantification. (Bottom) Photographs of AlpNPs suspensions: unwashed, after one wash, and after three washes. J) Quantitative analysis of residual PVA content in AlpNPs corresponding to (I). Data are mean ± SD (n=3). ^***^ *p* <0.001, one‐way ANOVA with Tukey's test.

In this study, AlpNPs were prepared using a solvent evaporation method, with acetone as the organic phase to dissolve PLGA and Alp, and the polyvinyl alcohol (PVA) as the outer aqueous phase (**Figure** **2**A,B). The resulting AlpNPs formed a colloidal suspension in PBS (Figure 2C). Transmission electron microscopy (TEM) images show that AlpNPs present a filamentous morphology, with an average particle size of 5.377×0.271 µm (Figure 2D). Dynamic light scattering (DLS) analysis showed that the average particle size of AlpNPs was 220.19 ± 30.34 nm, with a zeta potential of −23.57 ± 2.29 mV (Figure 2E,F). Stability is a significant factor responsible for the bioactivity and applications of nanomaterials. DLS analysis showed that with the increase of time, the particle size of AlpNPs did not change significantly (Figure S2A, Supporting Information). Furthermore, high‐performance liquid chromatography (HPLC) determined the encapsulation efficiency of AlpNPs to be 94.77% ± 1.68%. To simulate the in vivo drug release profile, the release kinetics of AlpNPs were evaluated in PBS at 37 °C. As shown in Figure 2G, compared to free Alp, AlpNPs exhibited a stable and sustained release over time, reaching a plateau around 12 h. UV–vis spectroscopy was performed to assess the absorption spectra of Alp, PLGA, and AlpNPs. Results demonstrated that PLGA exhibited no distinct absorption peaks, whereas Alp displayed a characteristic peak at 285 nm. Notably, AlpNPs showed increased absorbance at 285 nm and an additional absorption peak at 305 nm, indicating successful incorporation of Alp within PLGA (Figure 2H). To ensure that residual PVA from the preparation process was effectively removed, we quantified its concentration in the supernatant of AlpNPs following successive washes. The data (Figure 2I,J) show a dramatic reduction in PVA content from 789.4 ± 12.5 µg mL^−1^ (unwashed) to 1.19 ± 0.04 µg mL^−1^ (after three washes), validating that the purification process successfully strips PVA from the nanoparticle surface. Collectively, these findings confirm the successful design and synthesis of AlpNPs—nanoparticles encapsulating a natural compound—with promising potential for therapeutic intervention in AOH‐induced injury.

### 2.3 AlpNPs Promote M2‐Like Polarization of Microglia and Suppress Inflammation

**Figure 3:**
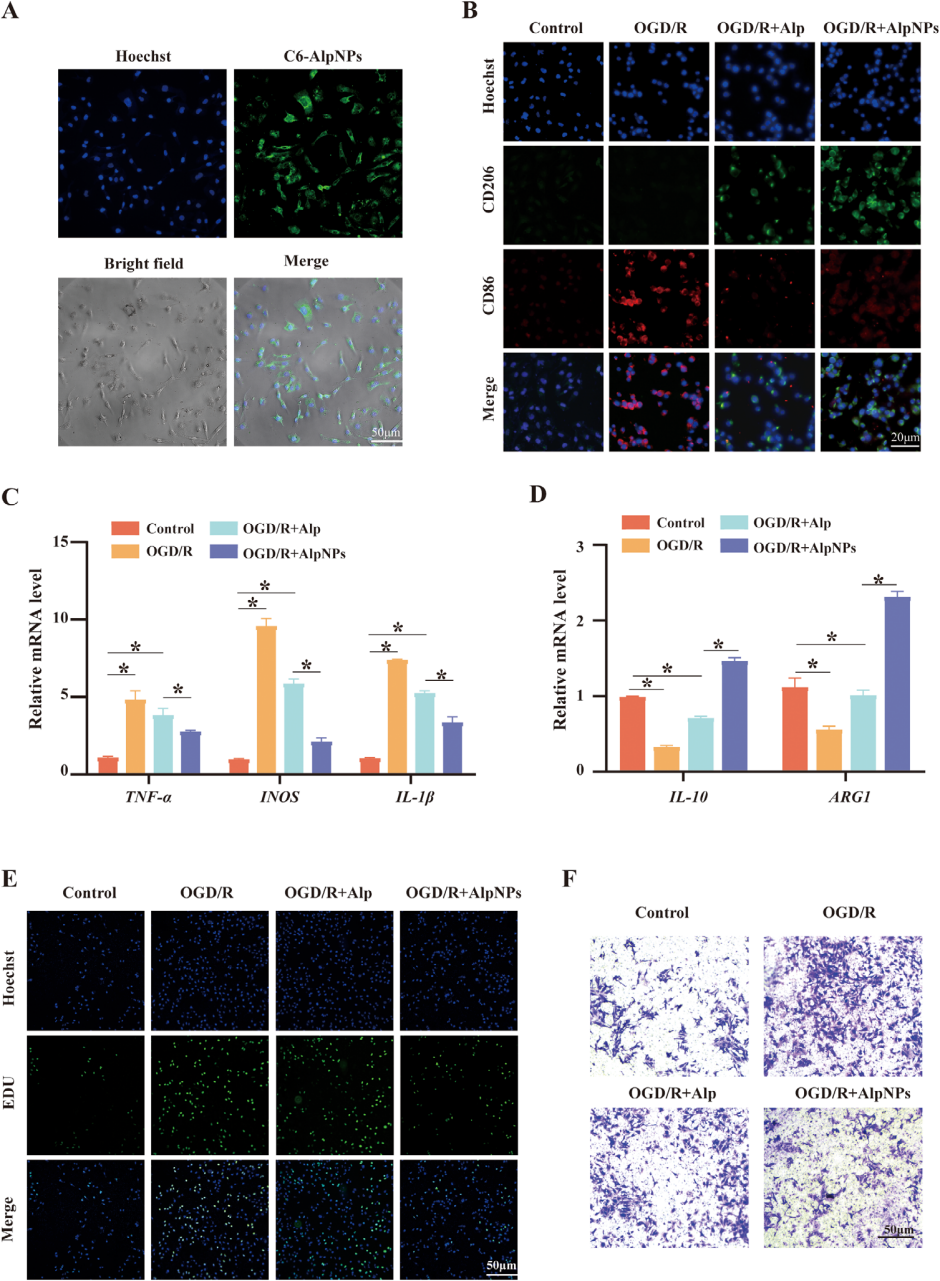
AlpNPs Promote M2 Polarization of Microglia and Suppress AOH Induced Neuroinflammation. A) Representative images showing the uptake of coumarin‐labeled AlpNPs (green fluorescence) by HMC3 microglial cells (scale bar = 50 µm). B) Immunofluorescence staining of CD86 (M1 marker) and CD206 (M2 marker) in HMC3 cells (scale bar = 20 µm). C,D) qPCR analysis of inflammatory and anti‐inflammatory cytokines. AlpNPs treatment reversed the upregulation of pro‐inflammatory genes and downregulation of anti‐inflammatory genes induced by OGD/R in HMC3 cells. E) Transwell assay assessing the migratory capacity of microglia in each group (scale bar = 50 µm). F) EDU assay evaluating the proliferative activity of HMC3 cells across the four experimental groups (scale bar = 50 µm). ^*^ *p* <0.05 was considered statistically significant.

Previous studies have demonstrated that the polarization of pro‐inflammatory microglia toward an anti‐inflammatory phenotype ameliorates the retinal inflammatory microenvironment and reduces RGCs death, underscoring the critical role of microglial anti‐inflammatory polarization in RGCs survival.^[^ ^28^ ^]^ In this study, using oxygen‐glucose deprivation/reoxygenation (OGD/R) treatment of microglia as an in vitro cell model, we further investigated the effects of AlpNPs on microglial physiological states. Firstly, the immortalized human microglial cell line human microglial clone 3 (HMC3), commonly used in neurodegenerative disease research, was employed to verify AlpNPs uptake in vitro. As shown in **Figure** **3**A, coumarin‐labeled AlpNPs (C6‐AlpNPs) with green fluorescence were effectively internalized by HMC3 cells and primarily localized within the cytoplasm, confirming successful nanoparticle uptake by microglia. HMC3 cells were co‐cultured with varying concentrations of free Alp (0, 25, 50, 100, and 200 µg mL^−1^) followed by OGD/R treatment, and cell viability was assessed via CCK‐8 assay. Cell viability increased in a dose‐dependent manner with Alp concentration, peaking at 100 µg mL^−1^. Notably, at this concentration, cells treated with AlpNPs exhibited significantly higher viability than those treated with free Alp (Figure S3A,B, Supporting Information). Furthermore, we found that AlpNPs at a concentration of 100 µg mL^−1^ exerted the most potent anti‐inflammatory effect (Figure S3C, Supporting Information). In contrast, treatment with PLGA nanoparticles alone did not affect microglial viability or reverse the inflammatory changes induced by OGD/R (Figure S3D,E, Supporting Information). Therefore, 100 µg mL^−1^ AlpNPs were selected to explore the neuroprotective effects against AOH‐induced microglial toxicity.

AOH exposure increased the proportion of microglia exhibiting an M1‐like pro‐inflammatory phenotype and elevated the release of inflammatory cytokines.^[^ ^29^ ^]^ To assess whether AlpNPs modulate microglial polarization, we evaluated the expression of M1 (CD86) and M2 (CD206) markers. As illustrated in Figure 3B, OGD/R treatment significantly upregulated M1 microglia, evidenced by increased green fluorescence intensity of CD86+ cells compared to controls. Treatment with AlpNPs reduced the number of CD86+ cells while enhancing the red fluorescence intensity of CD206+ cells, indicating a shift toward the M2 phenotype. Moreover, microglia pretreated with AlpNPs showed a marked attenuation of OGD/R‐induced increases in pro‐inflammatory mediators (*INOS, TNF‐α, IL‐1β*) alongside promotion of anti‐inflammatory mediators (*ARG1, IL‐10*) (Figure 3C,D). Consistent with the reactive state of microglia following injury, OGD/R treatment elicited a significant increase in microglial proliferation and migration, which was evidenced by EDU incorporation and Transwell assays.^[^ ^30^ ^]^ This enhanced response was effectively abolished by treatment with AlpNPs (Figure 3E,F). Consistent results were observed in primary microglia, where AlpNPs inhibited OGD/R‐induced inflammation and proliferation (Figure S4B–D, Supporting Information).

Collectively, these findings demonstrate that AlpNPs modulate microglial function in vitro by promoting the transition from a pro‐inflammatory M1 phenotype to an anti‐inflammatory M2 phenotype, concomitantly suppressing microglial proliferation and migration. This dual regulatory effect attenuates inflammatory responses and highlights the superior therapeutic potential of AlpNPs compared to free Alp in mitigating microglia‐mediated neuroinflammation.

### 2.4 AlpNPs Target LRP1‐PPARγ Axis to Alleviate Glaucoma‐Induced Lipid Accumulation

**Figure 4:**
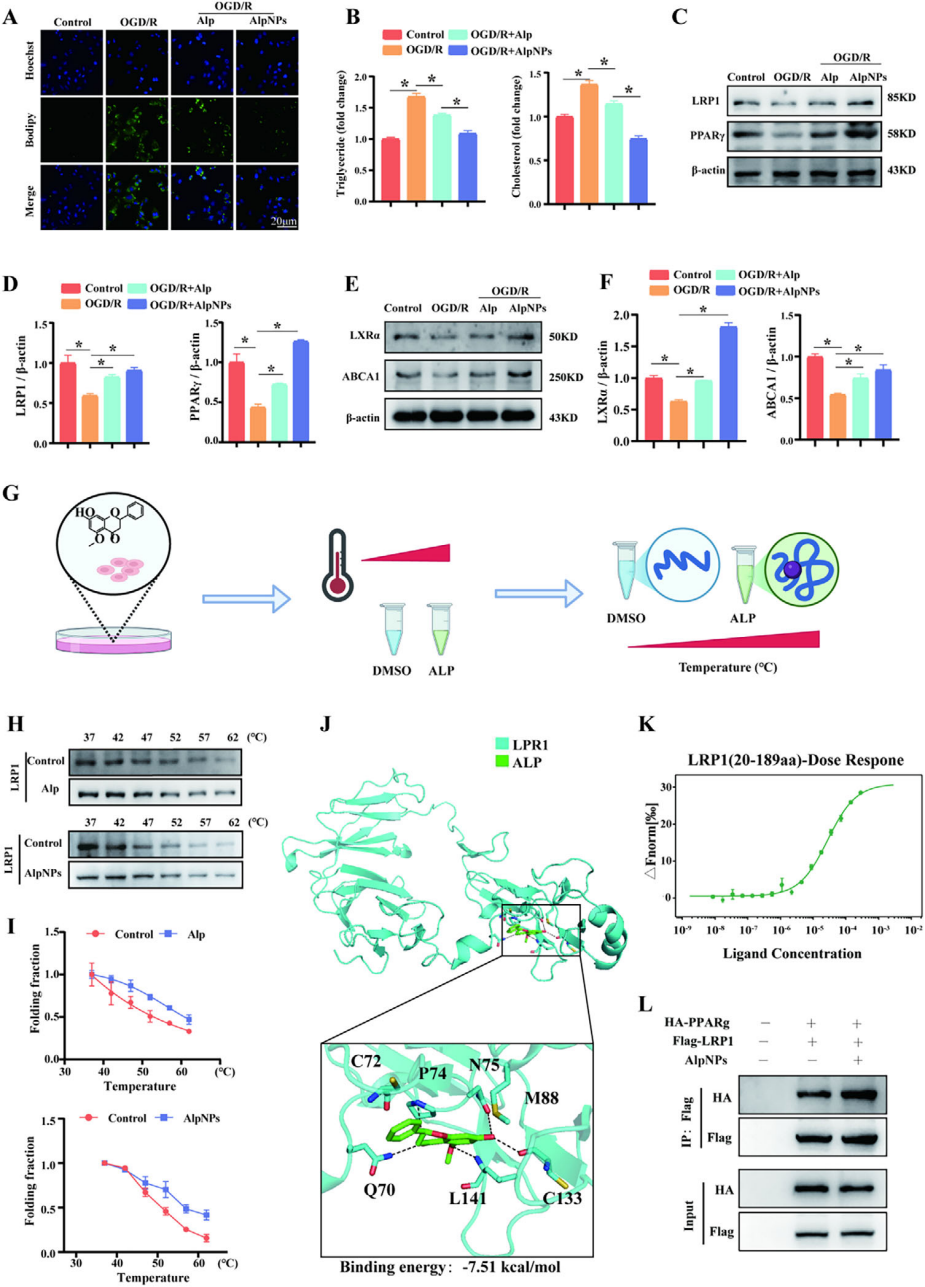
AlpNPs Target the LRP1‐PPARγ Pathway to Restore Lipid Metabolic Homeostasis in Microglia Following AOH. A) Immunofluorescence images of HMC3 microglial cells showing intracellular LD accumulation. AlpNPs treatment markedly reduced LD accumulation induced by OGD/R. Scale bar: 50 µm. B) Quantification of intracellular total cholesterol and triglyceride levels. AlpNPs significantly attenuated the OGD/R‐induced elevation of lipid content in microglia. C–F) Western blotting analysis of LRP1, PPARγ, LXRα, and ABCA1 protein expression in HMC3 cells treated with AlpNPs, along with corresponding quantification. β‐actin was used as a loading control. Data are presented as mean ± SD from three independent experiments. G) Schematic diagram of the CETSA workflow used to assess direct interaction between Alp and LRP1. H–I) CETSA‐based evaluation of LRP1 thermal stability following treatment with Alp or AlpNPs, conducted across a temperature range of 37 to 62 °C, indicating specific binding and stabilization of LRP1 by Alp. J) In silico molecular docking model predicting the interaction between Alp and LRP1. K) MST analysis confirmed a direct interaction between Alp and the LRP1 fragment (20‐189aa). L) Co‐IP analysis showing enhanced interaction between LRP1 and PPARγ in AlpNPs‐treated cells. Data are presented as mean ± SD. Statistical analyses were performed using Student's t‐test or one‐way ANOVA followed by Tukey's post hoc test. ^*^ *p* <0.05 was considered statistically significant.

Previous studies have demonstrated that Alp modulates lipid metabolism‐related genes to ameliorate non‐alcoholic fatty liver induced by a high‐fat diet.^[^ ^31^ ^]^ Alp also promotes cholesterol efflux via the PPARγ‐LXRα‐ABCA1 pathway, inhibiting ox‐LDL‐induced lipid accumulation in macrophages.^[^ ^32^ ^]^ LD accumulation in microglia is associated with dysfunction and pro‐inflammatory states in the brains of neurodegenerative disease models. To determine whether AlpNPs can reduce lipid accumulation, we measured LD levels in HMC3 cells using BODIPY 493/503 staining. AlpNPs significantly reduced LD accumulation in HMC3 cells compared to the OGD/R group (**Figure** **4**A), with consistent results observed in primary microglia (Figure S4E, Supporting Information). Furthermore, intracellular cholesterol and triglyceride levels were quantified using specific assay kits. AlpNPs significantly decreased total cholesterol levels in HMC3 cells (Figure 4B).

Based on these findings, we explored the molecular mechanisms underlying the neuroprotective effects of AlpNPs against AOH‐induced injury. Given the growing evidence implicating LRP1 in the pathophysiology of lipid metabolism, we investigated its role in the lipid‐regulating effects of Alp. The expression of lipid metabolism‐related proteins LRP1 and PPARγ was analyzed. As shown in Figure 4C–F, OGD/R treatment downregulated the protein expression of LRP1 and PPARγ, along with their downstream targets LXRα and ABCA1. Conversely, administration of AlpNPs effectively reversed these alterations. Similar trends were observed in primary microglia treated with AlpNPs (Figure S4F–I, Supporting Information).

To gain structural insights into how AlpNPs modulate the LRP1‐PPARγ pathway, we employed a combination of molecular docking, cellular thermal shift assay (CETSA), and microscale thermophoresis (MST) to determine if a direct interaction exists between Alp and LRP1. We first performed CETSA in HMC3 cells (Figure 4G), a method that assesses target engagement by measuring ligand‐induced changes in protein thermal stability.^[^ ^33^ ^]^ The results showed a significant stabilization of LRP1 across a temperature range of 37 °C to 62 °C following treatment with free Alp, and a similar stabilizing effect was observed with AlpNPs (Figure 4H,I). To confirm whether Alp directly binds to LRP1, molecular docking simulations were performed. The results revealed strong binding affinity between Alp and LRP1 (binding energy: −7.51 kcal mol^−1^). Molecular docking analysis revealed that Alp targets LRP1 through hydrogen bond interactions with residues Q70, P74, N75, C133, and L141, among which C133 exhibited the highest predicted binding affinity, suggesting its role as a critical binding site (Figure 4J). To functionally validate this prediction, we constructed an LRP1 C133A point mutant. CETSA demonstrated a marked reduction in the binding ability of AlpNPs to the mutant protein compared to the control group (Figure S5A,B, Supporting Information). Further biophysical validation was obtained using MST, which confirmed a direct interaction between Alp and a purified LRP1 fragment (20‐189aa), with a KD dissociation constant of 43.03 ± 0.22 µm (Figure 4K). Given prior evidence that LRP1 can directly bind the ligand‐binding domain of PPARγ to enhance its transcriptional activity and regulate lipid metabolism,^[^ ^16^ ^]^ we performed co‐immunoprecipitation (co‐IP) assays, which showed that AlpNPs strengthen the LRP1‐PPARγ interaction (Figure 4L). Together, these data provide strong evidence that AlpNPs act through direct binding to LRP1 promote its association with PPARγ, and subsequently upregulate LXRα and ABCA1 expression. This signaling axis facilitates cholesterol efflux, attenuates OGD/R‐induced microglial neurotoxicity, and unveils a novel mechanism by which AlpNPs ameliorate lipid dysregulation and neuroinflammation in glaucoma.

### 2.5 Knockdown of LRP1 Abolishes the Protective Effects of AlpNPs Against Glaucoma‐Induced Neuroinflammation and Lipid Accumulation in Microglia

**Figure 5:**
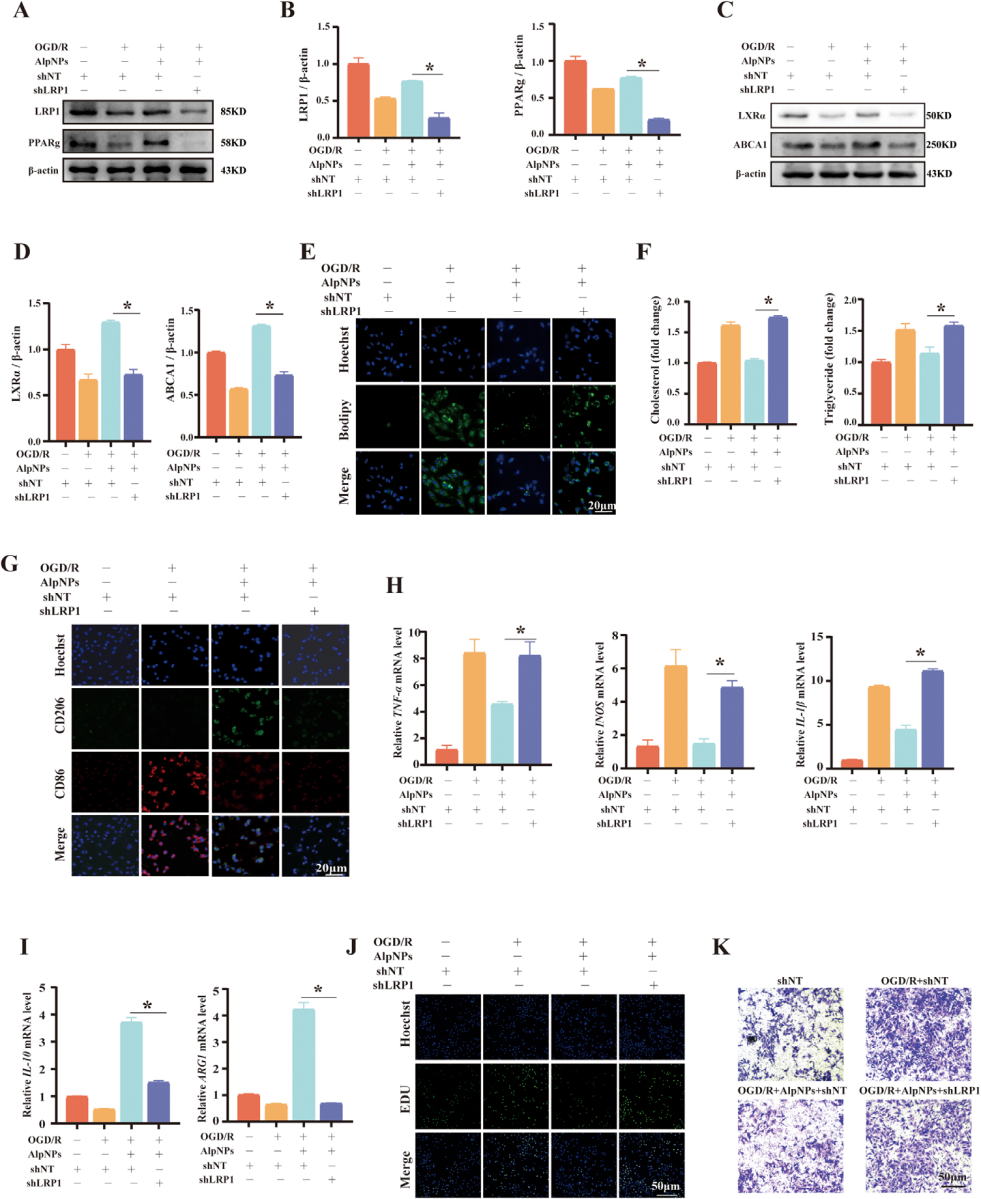
Knockdown of LRP1 Abrogates the Protective Effects of AlpNPs on Lipid Metabolism and Inflammatory Polarization in Microglia. A–D) Effect of LRP1 knockdown on protein expression in HMC3 microglial cells. (A,C) Representative western blotting images showing the protein levels of LRP1, PPARγ, LXRα, and ABCA1 following LRP1 knockdown. β‐actin was used as a loading control. (B,D) Densitometric quantification of the protein levels from (A,C), respectively, as analyzed by ImageJ. Data are presented as the mean ± SD from three independent experiments. E) Representative immunofluorescence images illustrating intracellular LD accumulation in HMC3 cells after LRP1 knockdown. Scale bar: 20 µm. F) Quantification of intracellular total cholesterol and triglyceride levels using commercial assay kits. G) Immunofluorescence staining of CD86 (M1 marker) and CD206 (M2 marker) in HMC3 cells under different treatment conditions. Scale bar: 20 µm. H,I) Bar graphs showing mRNA expression levels of pro‐inflammatory (*INOS, TNF‐α, IL‐1β*) and anti‐inflammatory (*ARG1, IL‐10*) cytokines under various conditions, as determined by qPCR. (K) EDU incorporation assay assessing cell proliferation in HMC3 microglia. Scale bar: 50 µm. J) Representative images of transwell migration assays evaluating the effect of different treatments on HMC3 cell migration. Scale bar: 50 µm. ^*^ *p* <0.05 was considered statistically significant.

To further elucidate the role of LRP1 in this process, we reduced LRP1 expression using shRNA‐mediated knockdown. HMC3 cells were transfected with two distinct LRP1 shRNAs (shRNA#1 and shRNA#2). The results demonstrated that both shRNAs significantly reduced LRP1 protein levels compared to the control group, with shRNA#2 being the most effective and subsequently selected for further experiments (Figure S6A–C, Supporting Information). We next investigated whether LRP1 knockdown (shLRP1) alters the protective effects of AlpNPs against OGD/R‐induced neuroinflammation. In HMC3 cells with LRP1 knockdown, the protein expression of LRP1, PPARγ, LXRα, and ABCA1 remained significantly suppressed under OGD/R conditions, and this suppression was not rescued by AlpNPs treatment (**Figure** **5**A–D). Furthermore, shLRP1 transfection exacerbated LD accumulation and increased intracellular total cholesterol and triglyceride levels in HMC3 cells pre‐treated with AlpNPs during OGD/R‐induced abnormal lipid metabolism (Figure 5E,F). These findings indicate that shLRP1 transfection effectively blocked the protective effects of AlpNPs on OGD/R‐induced lipid metabolic dysfunction. The shLRP1‐induced accumulation of LD highlights that AlpNPs exert their protective effects through LRP1 signaling.

Additionally, immunofluorescence analysis revealed that shLRP1 transfection reduced the number of CD206+ cells, accompanied by enhanced red fluorescence intensity indicative of CD86+ cells, during OGD/R‐induced neurotoxicity compared to cells pre‐treated with AlpNPs (Figure 5G). Consistently, during OGD/R‐induced neuroinflammation, shLRP1 transfection increased the mRNA expression levels of pro‐inflammatory mediators (*INOS, TNF‐α, IL‐1β*) and decreased the expression of anti‐inflammatory mediators (*ARG1, IL‐10*) in AlpNPs‐pretreated HMC3 cells (Figure 5H,I). Regarding other microglial functions, EDU assays demonstrated that LRP1 knockdown influenced microglial proliferation (Figure 5J). Similarly, transwell assays showed a significant increase in microglial migration in the OGD/R+AlpNPs+shLRP1 group (Figure 5K).

In summary, LRP1 knockdown partially abrogates the protective effects of AlpNPs against OGD/R‐induced neuroinflammation by impairing M2‐like polarization of microglia. This underscores the critical role of LRP1 in mediating AlpNPs' neuroprotective effects.

### 2.6 Microglial LRP1 Knockdown Reverses the Protective Effects of AlpNPs on RGCs

**Figure 6:**
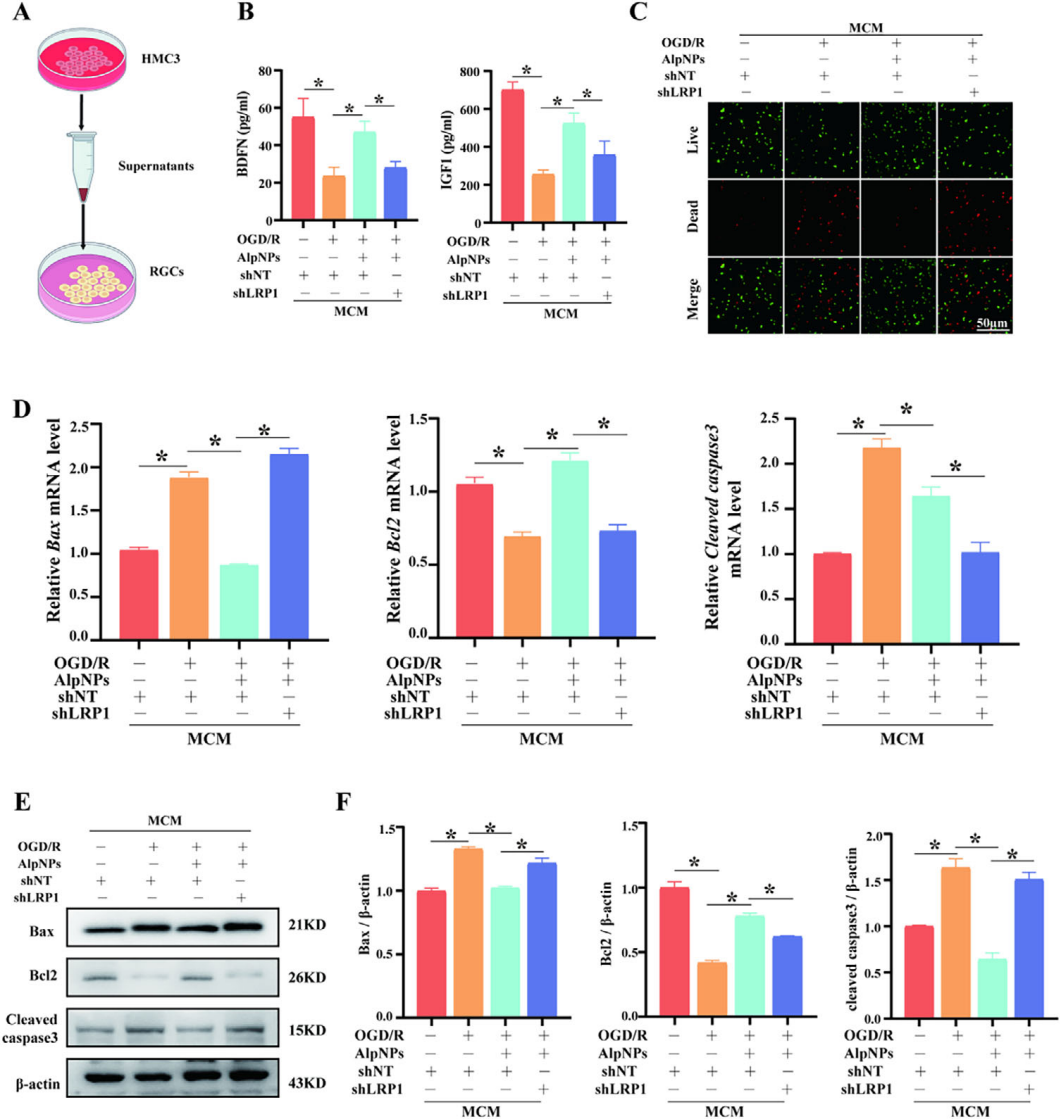
Effects of Microglial LRP1 Knockdown on the Survival of Primary RGCs. A) Schematic illustration of the co‐culture experimental design involving microglia and RGCs. B) The contents of BDNF and IGF‐1 in the conditioned medium were detected by ELISA kits. C) Representative live/dead staining images of RGCs co‐cultured with conditioned media from microglia treated with shLRP1 and/or AlpNPs. Live cells are shown in green; dead cells are shown in red. Scale bar: 50 µm. D) Bar graph showing mRNA expression levels of apoptosis‐related genes *Bax, Bcl2*, and *Cleaved caspase‐3* under different treatment conditions. E,F) Quantification of protein expression levels of Bax, Bcl2, and Cleaved caspase‐3, assessed by western blotting. ^*^ *p* <0.05 was considered statistically significant.

Studies have demonstrated that M1‐type microglia secrete increased levels of pro‐inflammatory cytokines and chemokines, which not only directly damage retinal neurons but also exacerbate inflammation by recruiting circulating immune cells. To investigate whether activated microglia damage RGCs under pathological conditions and to evaluate the role of AlpNPs in this process, we isolated and cultured primary RGCs for subsequent experiments. As shown in **Figure** **6**A, we conducted co‐culture experiments of microglia and RGCs.

Compared to microglia‐conditioned medium (MCM) (shNT), the supernatant from OGD/R‐stimulated microglia (MCM (OGD/R)) significantly induced primary RGCs death. However, treatment with Alp or AlpNPs provided protection to the RGCs, with AlpNPs exhibiting superior efficacy (Figure S7B–E, Supporting Information). To investigate whether AlpNPs promote RGCs survival by stimulating the secretion of neurotrophic factors from M2‐polarized microglia in an LRP1‐dependent manner, we measured the levels of brain‐derived neurotrophic factor (BDNF) and insulin‐like growth factor 1 (IGF‐1) in the conditioned medium by ELISA. The results showed that compared to the conditioned medium from the MCM(shNT+OGD/R) group, treatment with AlpNPs significantly increased the secretion of BDNF and IGF‐1. This was concomitant with enhanced RGCs survival, upregulated Bcl2 expression, and downregulated Bax expression (Figure 6B–F). Crucially, these therapeutic effects were abolished upon LRP1 knockdown. In summary, AlpNPs enhance the secretion of neurotrophic factors by promoting LRP1‐dependent M2 microglial polarization, thereby attenuating RGCs death. These findings underscore the pivotal role of LRP1 in mediating the neuroprotective effects of AlpNPs.

### 2.7 AlpNPs Alleviate Lipid Accumulation and RGCs Death Induced by Acute Glaucoma

Prior to evaluating the protective effects of AlpNPs in the AOH model, we first assessed their distribution following intravitreal injection. To determine the bio‐distribution of AlpNPs in the retina, coumarin, a fluorescent dye, was covalently conjugated to AlpNPs.^[^ ^34^ ^]^ These C6‐AlpNPs were administered via intravitreal injection one day before AOH induction, and retinal distribution was assessed 3 days post‐injection. As shown in Figure S8A,B (Supporting Information), AlpNPs were dispersed throughout the retinal layers. Critically, we observed a partial colocalization of the C6 signal with Iba1‐positive microglia, evidenced by distinct green fluorescent puncta surrounding and within these immune cells. This fluorescent signal was notably present at the 3‐day time point but had largely diminished by the second week (Figure S8C, Supporting Information), suggesting that AlpNPs initially accumulate near and are potentially internalized by microglia before being cleared over time. To complement this spatial analysis, we quantitatively measured AlpNPs retention in the retina using HPLC at 1‐, 3‐, 7‐, 14‐, and 30‐days post‐injection. As shown in Figure S8D (Supporting Information), the retinal concentration of AlpNPs was maximal on day 1 post‐injection. By days 7, 14, and 30, only minimal levels of AlpNPs remained detectable.

We next conducted a comprehensive biosafety assessment of AlpNPs. Histological analysis of retinal sections revealed no significant morphological alterations following intravitreal injection of AlpNPs compared to the normal control group (Figure S9A, Supporting Information). Evaluation of hepatorenal function, based on plasma levels of alanine aminotransferase (Alt), aspartate aminotransferase (Ast), and uric acid (Ua), showed no marked changes, indicating an absence of detectable systemic toxicity (Figure S9B, Supporting Information). Consistent with these findings, histopathological examination of major organs (heart, liver, and kidney) via H&E staining revealed no obvious lesions (Figure S9C, Supporting Information). Collectively, these results demonstrate the favorable biosafety profile of AlpNPs for intraocular application.

**Figure 7:**
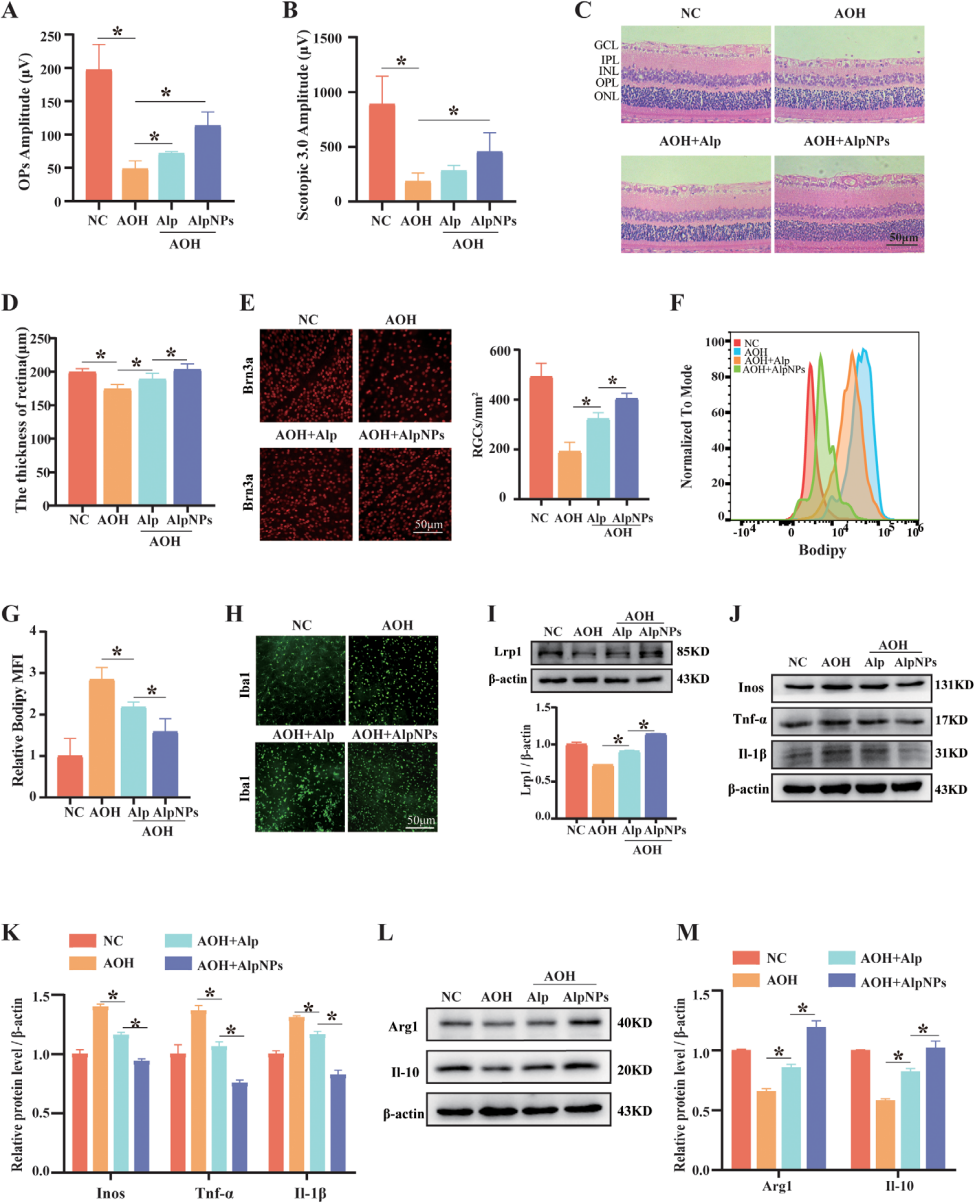
AlpNPs Effectively Alleviate RGCs Damage in the AOH Model. A,B) The amplitudes of 3.0cd's m^−2^ and OPs are quantified. C,D) Representative H&E stained images of retinal sections from each group (*n* = 5). Scale bar: 50 µm. E) Quantification of RGCs numbers in the retinas of different treatment groups (*n* = 5). Scale bar: 50 µm. F,G) Flow cytometric analysis of mean fluorescence intensity of LD in the retina (*n* = 3). H) Representative western blotting image and corresponding quantification of Lrp1 expression in total retinal protein extracts. I) Morphological analysis of Iba1⁺ microglia in retinal sections (*n* = 4). Scale bar: 50 µm. J–M) Western blotting analysis of pro‐inflammatory and anti‐inflammatory protein expression levels in the retina. ^*^ *p* <0.05 was considered statistically significant.

Subsequently, we investigated the therapeutic efficacy of AlpNPs against AOH‐induced optic nerve injury. To evaluate retinal visual function, we performed electroretinography (ERG) after overnight dark adaptation.^[^ ^35^ ^]^ Quantitative analysis demonstrated that the AlpNPs‐treated group exhibited a significant recovery in both the amplitude of the scotopic 3.0 and the oscillatory potentials (OPs) by day 3 post‐injection compared to the AOH group (**Figure** **7**A,B), a finding supported by representative waveform traces (Figure S10A,B, Supporting Information). This functional improvement was corroborated by histological analysis. H&E staining revealed that the AOH+AlpNPs group showed only mild retinal thinning and disorganization compared to the severe damage in the AOH group (Figure 7C,D). Furthermore, quantification of surviving RGCs confirmed that AlpNPs treatment significantly attenuated AOH‐induced RGCs death (Figure 7E). Furthermore, LD fluorescence intensity in retinal cells was significantly reduced following AlpNPs treatment (Figure 7F,G). Morphological analysis of retinal whole mounts stained for lipid deposits yielded concordant results (Figure S10C, Supporting Information), providing visual evidence of the attenuated lipid accumulation. At the single‐cell level, morphological analysis of microglia processes was performed. Acute ocular hypertension significantly increased the fluorescence intensity and the same size of Iba1+ microglia while decreasing their total process length and branch numbers. These alterations were partially reversed by AlpNPs treatment (Figure 7H). Lastly, we evaluated the protein levels of LRP1 and inflammatory factors in Sprague‐Dawley (SD) rats. Compared to the AOH group, the AOH+AlpNPs group showed elevated Lrp1 protein and anti‐inflammatory factor levels while pro‐inflammatory factor levels were reduced (Figure 7I–M). In conclusion, our data demonstrates that AlpNPs effectively suppress microglial polarization and mitigate optic nerve damage induced by acute glaucoma.

## 3 Discussion

Glaucoma is a leading cause of irreversible blindness worldwide, characterized by the progressive loss of RGCs and degeneration of the optic nerve. While elevated IOP is the most recognized risk factor and therapeutic target, a substantial proportion of patients continue to experience visual field deterioration despite effective IOP control. This clinical dilemma has prompted increasing interest in IOP‐independent mechanisms, including neuroinflammation, glial dysfunction, and metabolic reprogramming, as key contributors to glaucomatous neurodegeneration.^[^ ^4^, ^36^, ^37^ ^]^ Among these, microglial dysregulation plays a central role in shaping the retinal immune microenvironment, modulating neuronal survival, and dictating the course of disease progression.^[^ ^38^, ^39^ ^]^

Our study provides compelling evidence that microglial lipid metabolic dysfunction is a key pathogenic event in glaucoma. Using an AOH model, we demonstrated that retinal microglia exhibit pronounced LD accumulation, a shift toward proinflammatory M1‐like polarization, and reduced expression of the lipid transporter LRP1. Notably, transcriptomic analysis also revealed alterations in other metabolic regulators such as VAPB. The observed mRNA‐protein discrepancy for VAPB may reflect post‐transcriptional regulatory mechanisms, a phenomenon often associated with cellular stress responses. This lipid‐accumulating microglial phenotype mirrors findings in other neurodegenerative conditions, such as Alzheimer's disease, where impaired lipid metabolism exacerbates neuroinflammation and compromises phagocytic function.^[^ ^10^, ^40^, ^41^ ^]^ Similarly, our results suggest that lipid overload in microglia not only disrupts metabolic homeostasis but also fosters a neurotoxic microenvironment that accelerates RGCs loss.

It is noteworthy that our study employed intravitreal injection for AlpNPs delivery, an established local administration strategy in retinal therapeutics. While this approach enables precise intraocular drug delivery and is widely used in both preclinical and clinical settings—including in recent innovative nanoparticle‐based therapies for neuroprotection and microglial modulation^[^ ^42^, ^43^ ^]^—it does carry potential risks such as infection, inflammation, and elevated intraocular pressure. We fully recognize that repeated invasive procedures may pose challenges for the long‐term management of chronic conditions like glaucoma. Importantly, recent advances in non‐invasive strategies—such as nanoparticle‐based eye drops, sustained‐release hydrogels, and iontophoresis—have shown promising potential in improving patient compliance and reducing complications.^[^ ^44^, ^45^ ^]^ In the present study, intravitreal injection was primarily adopted to ensure sufficient AlpNPs delivery and reliable mechanistic validation during this proof‐of‐concept stage. We emphasize that future clinical translation should prioritize the development of non‐invasive or long‐acting sustained‐release alternatives. Thus, while our findings establish the efficacy of AlpNPs via local administration, they also lay a solid foundation for developing more clinically adaptable formulations in the future.

Notably, transcriptomic analyses of glaucoma‐related GEO datasets revealed consistent downregulation of Lrp1, and its decreased expression was further validated in both AOH retina and serum samples from glaucoma patients. This human‐animal concordance underscores the translational relevance of our findings. LRP1 has been previously implicated in cholesterol homeostasis, apoptotic cell clearance, and suppression of inflammatory cascades, particularly through interaction with nuclear receptors such as PPARγ.^[^ ^16^, ^46^ ^]^ However, its role in retinal microglia has remained largely unexplored. Our study bridges this gap, identifying LRP1 not only as a key metabolic regulator but also as a central modulator of microglial polarization in the glaucomatous retina.

To therapeutically address this dysfunction, we developed a nanomedicine approach by encapsulating the natural flavonoid Alp into biodegradable PLGA nanoparticles. This strategy aligns with recent advances in nanoparticle‐based therapies for neuroprotection and microglial modulation,^[^ ^42^, ^43^, ^47^ ^]^ which demonstrate the growing potential of nanomedicine in treating neurological and ocular disorders. While Alp is known for its anti‐inflammatory and metabolic benefits, its clinical use is limited by poor solubility and rapid metabolism. The nanoparticle formulation markedly enhanced its pharmacokinetic profile, enabled targeted delivery to microglia, and improved its therapeutic efficacy. Compared to free Alp, AlpNPs showed superior capacity to reduce LD accumulation, restore lipid metabolic balance, promote M2‐like polarization, and suppress proinflammatory cytokine production. These findings are consistent with prior studies showing that nanoformulations of bioactive compounds like curcumin and resveratrol improve cellular uptake, tissue penetration, and therapeutic outcomes in CNS disorders.^[^ ^48^, ^49^ ^]^

Mechanistically, we demonstrated that AlpNPs exert their protective effects through direct engagement of the LRP1‐PPARγ axis. Molecular docking and CETSA confirmed that Alp binds directly to LRP1, stabilizing the protein and enhancing its interaction with PPARγ. **To further solidify this signaling cascade, we investigated key downstream effectors of PPARγ. We found that AlpNPs treatment significantly upregulated the protein levels of LXRα and its target gene, ABCA1, which are well‐established mediators of PPARγ’s functions in lipid metabolism and inflammation. LXRα is a master regulator of cholesterol efflux, and ABCA1 plays a critical role in transporting cholesterol to apolipoproteins. The activation of this PPARγ‐LXRα‐ABCA1 axis provides a direct mechanistic explanation for the observed reduction in LD and the promotion of cholesterol homeostasis in microglia. This finding strongly supports that AlpNPs not only initiate the LRP1‐PPARγ interaction but also successfully trigger the entire functional downstream pathway responsible for lipid clearance and anti‐inflammatory responses**. This activation leads to the upregulation of lipid efflux genes, suppression of inflammatory mediators, and reprogramming of microglial immune‐metabolic state toward a neuroprotective phenotype. Previous research has shown that PPARγ agonists such as pioglitazone ameliorate neuroinflammation in multiple sclerosis and AD models by similar mechanisms.^[^ ^50^, ^51^ ^]^ However, our study is the first to demonstrate that a natural compound in nanoparticle form can modulate this pathway specifically in retinal microglia and in the context of glaucoma. LRP1 knockdown abolished the therapeutic effects of AlpNPs, confirming its essential role in mediating neuroprotection. These findings are consistent with reports that LRP1 deficiency exacerbates neuroinflammation in other injury and degeneration models.^[^ ^52^, ^53^, ^54^ ^]^ Therefore, our data thus establish a direct link between microglial lipid metabolism, immune activation, and RGC loss in glaucoma.

Clinically, AlpNPs offer several advantages. The PLGA matrix is FDA‐approved and has been widely used in ocular drug delivery systems.^[^ ^55^ ^]^ The observed sustained release kinetics and specific localization to Iba1+ microglia following intravitreal injection suggest that AlpNPs could reduce injection frequency and enhance targeted efficacy. Moreover, our results in patient serum samples suggest that LRP1 could serve as a potential biomarker for disease activity and therapeutic response. Nevertheless, further studies are needed to validate the safety and efficacy of AlpNPs in large‐animal models and assess potential off‐target effects. **Despite the promising results, several limitations should be acknowledged. First, the use of an acute AOH model, while a limitation for modeling chronic disease, was strategic for this initial mechanistic study. It allowed for rapid, reproducible induction of pathology, ideal for establishing causality and evaluating acute treatment efficacy. Future studies will essentiallly transition to chronic models to assess long‐term outcomes**. Second, although our study focused on microglia, other glial cells such as Müller cells and astrocytes may also be influenced by metabolic reprogramming and warrant further investigation. Third, we focused primarily on the LRP1‐PPARγ axis, but it is possible that AlpNPs modulate additional pathways such as AMPK, NRF2, or SIRT1, which are known to regulate glial metabolism and inflammation.

**Figure 8:**
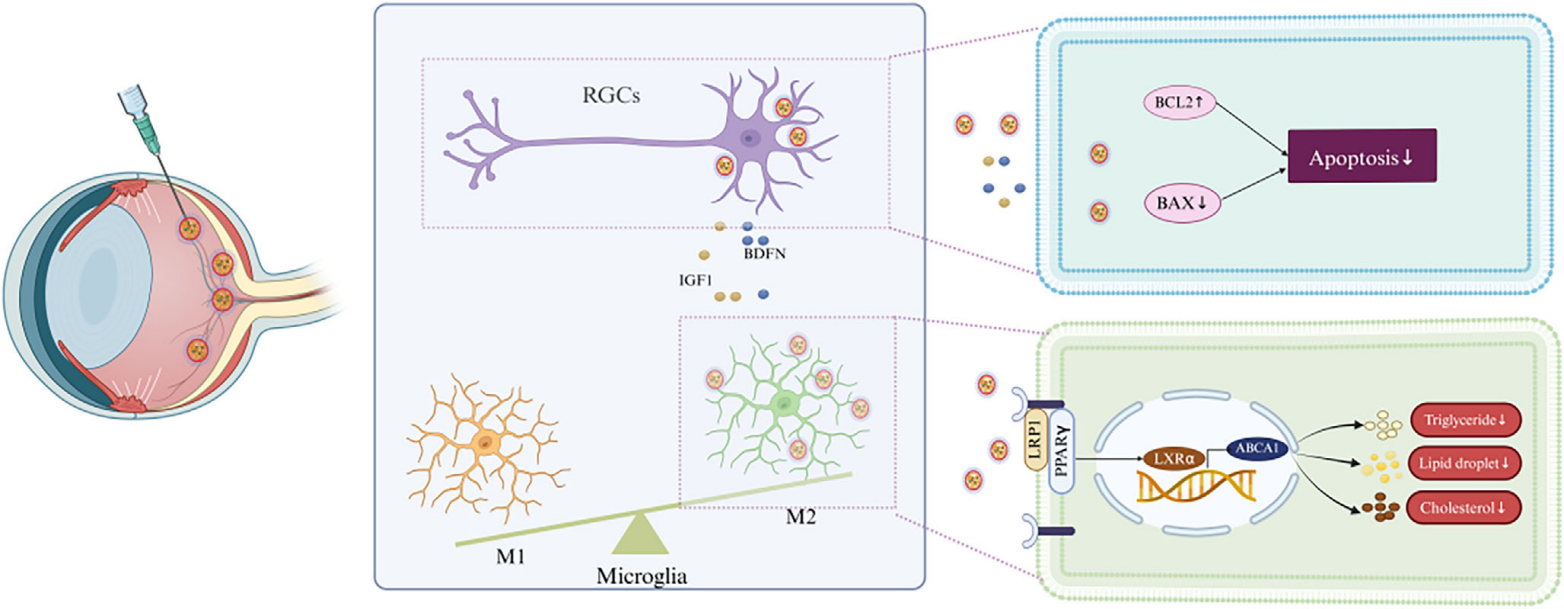
AlpNPs Promote M2‐like Polarization of Microglia and Target the LRP1‐PPARγ Signaling Pathway to Reduce Lipid Accumulation, Alleviating RGCs Injury Induced by Acute Glaucoma.

In summary, our study identifies microglial lipid metabolic dysfunction as a critical driver of glaucoma pathogenesis and presents AlpNPs as an effective nanotherapeutic strategy. By specifically targeting and binding to the LRP1 receptor on microglia, AlpNPs activate the LRP1‐PPARγ signaling axis, thereby restoring lipid metabolic homeostasis, promoting microglial polarization toward a neuroprotective M2 phenotype, and suppressing neuroinflammatory responses. These effects collectively lead to a marked attenuation of optic nerve injury and robust protection of RGCs (**Figure** **8**). These findings provide a novel mechanistic framework for future neuroprotective interventions in glaucoma and related neurodegenerative disorders.

## 4 Conclusion

In conclusion, this study establishes microglial lipid metabolic dysfunction as a key mechanism in glaucoma pathogenesis. Using an AOH model, we demonstrated that microglia exhibit abnormal LD accumulation, LRP1 downregulation, and a pronounced shift toward a pro‐inflammatory M1 phenotype. To counteract these pathological changes, we developed a novel AlpNPs delivery system that significantly improved drug bioavailability and microglial targeting. AlpNPs not only alleviated lipid accumulation and restored LRP1 expression but also promoted anti‐inflammatory M2 polarization, suppressed microglial proliferation and migration, and effectively protected RGCs from glaucomatous damage. Mechanistically, AlpNPs act through the LRP1‐PPARγ axis, activating downstream targets LXRα and ABCA1 to restore metabolic and immune balance. The abolition of these effects upon LRP1 knockdown confirms its central role. Our findings underscore the therapeutic potential of targeting microglial lipid metabolism via LRP1‐PPARγ signaling in glaucoma and possibly other neurodegenerative diseases.

## 5 Experimental Section

### Animals

SD neonatal and 6–8‐week‐old adult SD were procured from the Experimental Animal Center of Chongqing Medical University. Neonatal rats served as the source for primary microglia and RGCs cultures, whereas adult rats were employed to establish the AOH model. All animals were housed under standard laboratory conditions and handled in accordance with the NIH Guide for the Care and Use of Laboratory Animals (License No. SYXK (Yu) 2022‐0016). All procedures conformed to the ARVO Statement for the Use of Animals in Ophthalmic and Vision Research and were approved by the Institutional Animal Care and Use Committee of Chongqing Medical University (IACUC‐CQMU‐2024‐11020).

### AOH Model Establishment and Intravitreal Injection

The AOH model was widely accepted as an effective tool to simulate acute glaucomatous injury. The procedures for intravitreal injection and AOH model induction were performed as previously described.^[^ ^56^ ^]^ Briefly, 2 µL of Alp or AlpNPs at a concentration of 10 mg mL^−1^ were injected into the vitreous cavity of the right eye, while the left eye received 2 µL of PBS as a control. 24 h after the intravitreal injection, the AOH model was established.

Specifically, 6‐8‐week‐old adult SD rats were anesthetized by intraperitoneal injection of 1.25% avertin at a dose of 10 mL kg^−1^. Pupillary dilation and topical anesthesia were induced prior to the procedure. A 30‐gauge infusion needle, connected to a balanced salt solution reservoir elevated to 1.5 meters, was inserted into the anterior chamber of the right eye and maintained in place for 1 h, carefully avoiding injury to the corneal endothelium, iris, and lens. IOP was measured six times during this period using a TonoLab tonometer. The development of retinal pallor was observed, indicating retinal ischemia due to impaired blood supply. The contralateral eye underwent sham surgery without IOP elevation and served as the control.

### Bioinformatics Analysis

Expression profile data of glaucomatous retinal tissue analyzed in this study were obtained from the GEO at GSE130610, GSE192509, and GSE43671. To identify DEGs associated with glaucoma and lipid droplet‐related processes, we analyzed each dataset using the RStudio (RRID:SCR_000432) with the criteria of |logFC| > 0.5 and *p* <0.05. Subsequently, the DEGs were intersected with lipid droplet‐related genes, including those involved in lipid metabolism and cholesterol regulation, to obtain the key genes implicated in lipid metabolic dysregulation in glaucoma.

### Study Participants

This study enrolled 52 subjects from the Department of Ophthalmology at the First Affiliated Hospital of Chongqing Medical University, including 17 patients with POAG combined with ARC, 19 patients with PACG combined with ARC, and 16 patients with ARC alone. Serum samples (1 mL per patient, total 55 samples) were collected and the concentrations of LRP1 and VAPB in serum were measured using ELISA kits (HB2843, HB4105, Hengyuan Biotechnology Co., LTD., China) according to the manufacturer's protocols. Patients with known coronary heart disease, diabetes, or hypertension were excluded. All procedures were conducted with informed consent and approved by the Ethics Committee of the First Affiliated Hospital of Chongqing Medical University (approval number: 2024‐098‐02). The study strictly adhered to the principles of the Declaration of Helsinki.

### H&E Staining

All animals were euthanized at predefined time points, and eyes were promptly enucleated and fixed in FAS ocular fixative (G1109, Servicebio, China). Following graded ethanol dehydration, tissues were paraffin‐embedded using standard protocols. Sections (4 µm thick) were cut, deparaffinized, and stained with H&E. Four regions located 1 mm from the optic disc were selected for evaluation. Retinal thickness was measured using ImageJ (RRID:SCR_0 03070) by two independent, blinded observers in duplicate.^[^ ^57^ ^]^

### Synthesis of AlpNPs

Briefly, 1 mg of Alp (HY‐N0625A, MedChemExpress, USA) and 5 mg of PLGA (40 KDa, T19521, TargetMol, USA) were dissolved in 1 mL of acetone by ultrasonication for 5 min to form the organic phase. Using a 1 mL syringe, the organic phase was dropwise added at a constant rate into 1% polyvinyl alcohol (PVA; P139537, Aladdin, China) aqueous solution under continuous ultrasonication with a cell ultrasonic disruptor for 5 min to produce a milky oil‐in‐water (O/W) emulsion. The organic solvent was subsequently removed by rotary evaporation for 6 h. The resulting nanoparticle suspension was centrifuged and washed three times with deionized water at 15,000 rpm for 10 min at 4 °C to obtain the AlpNPs.

### Characterization of AlpNPs

The AlpNPs were characterized using multiple analytical techniques. Morphological examination was performed by transmission electron microscopy (RRID:SCR_02 5398) following sample preparation involving dispersion in PBS, deposition onto copper grids, and air‐drying. Colloidal stability was assessed through dynamic light scattering and zeta potential measurements using a Malvern Panalytical Zetasizer Nano system (RRID:SCR_02 5760), with samples prepared by sonication in PBS and analyzed in triplicate. UV–vis spectral analysis was conducted using an Agilent Cary 8454 UV–vis Diode Array System (RRID:SCR_01 9486) after dissolving PLGA, Alp, and AlpNPs in methanol. Encapsulation efficiency was determined by high‐performance liquid chromatography (Shimadzu Nexera HPLC/UHPLC Pump – LC‐40D XS, RRID:SCR_02 6528) using a standard curve generated from Alp solutions (5–160 µg mL^−1^), with AlpNPs degraded in methanol via sonication prior to analysis. In vitro release profiling was performed by placing equal amounts of free Alp and AlpNPs (1 mg) in dialysis bags immersed in PBS with 10% SDS, followed by incubation at 37 °C with agitation and sample collection at predetermined intervals over 36 h for HPLC analysis.

### Biosafety Evaluation

The in vivo biosafety of AlpNPs was systematically evaluated. Rats received an intravitreal injection of AlpNPs and were euthanized on days 0, 3, 7, and 14 post‐injections. Major organs, including the eyes, heart, liver, and kidneys, were harvested for histopathological examination using H&E staining. Additionally, serum levels of Ast, Alt, and Ua were measured to assess potential hepatorenal toxicity.

### Study on the In Vivo Distribution and Pharmacokinetics of AlpNPs

To comprehensively evaluate the intraocular distribution and metabolism of AlpNPs, this study integrated spatial localization and temporal dynamic analyses. The distribution of AlpNPs was visualized via fluorescence microscopy in retinal cryosections at 3 days after a single intravitreal injection. Concurrently, the pharmacokinetic profile was characterized by measuring alpinetin concentrations in retinal tissues at 1‐, 3‐, 7‐, 14‐, and 30‐days post‐injection using HPLC, allowing for the construction of a concentration‐time curve.

### ERG

Prior to recording, all animals were dark‐adapted overnight. Under dim red light, rats were anesthetized and pupils were dilated. Electrodes were positioned as follows: a ground electrode on the tail, reference electrodes beneath each eye, and active electrodes on the corneal surface. Scotopic ERG responses were recorded using a visual electrophysiology system (GanzfeldQ450SC Electrophysiological Test Unit (RRID:SCR_02 7489)) at flash intensities of 0.01, 3.0, and 10.0 cd·s m^−^^2^. All recordings were obtained during a single session. The amplitudes of the a‐wave, b‐wave, and OPs were analyzed.

### Cell Culture and Treatment

Primary microglia and RGCs were isolated from one‐day‐old SD rat pups following the protocol previously described.^[^ ^58^ ^]^ Primary microglia were identified by immunocytochemical staining using the Iba1 antibody (Figure S4A, Supporting Information), while primary retinal neurons were confirmed by Brn3a antibody staining (Figure S7A, Supporting Information). Fluorescence microscopy (Leica, Wetzlar, Germany) was employed to randomly select six fields per culture dish for quantification of positive cells. In addition, a HMC3 cell line (CLS Cat# 300 102, RRID: CVCL_II76) was utilized. HMC3 cells were authenticated by short tandem repeat (STR) profiling and confirmed to be free of mycoplasma contamination. Cells were maintained in MEM supplemented with 10% fetal bovine serum (FBS) and 1% penicillin‐streptomycin under standard culture conditions (37 °C, 5% CO_2_).

To simulate the ischemia‐reperfusion conditions of the AOH model in vitro, both HMC3 cells and primary microglia were pretreated with Alp or AlpNPs for 6 h. Subsequently, cells were transferred to glucose‐free medium and incubated under hypoxic conditions (5% CO_2_ and 95% N_2_) at 37 °C for an additional 6 h. Reperfusion was simulated by returning the cells to normoxic culture conditions with complete medium for 24 h.

### Cell Transfection

The LRP1 coding sequence was cloned onto the PCDH‐Flag vector to construct the expression plasmids of LRP1 and LRP1 C133A mutant, and the expression plasmid of PPARγ protein was constructed on the PCDH‐HA vector. Expression efficiency was confirmed by Western blot analysis. Plasmid construction was performed using the ClonExpress II One Step Cloning Kit (C112, Vazyme, China). Short hairpin RNA constructs targeting LRP1 (shLRP1#1 and shLRP1#2) were cloned into the pLKO.1 vector. Transfections were carried out using Lipofectamine™ 3000 reagent (L3000‐015, Thermo Fisher Scientific, USA) according to the manufacturer's instructions. The sequences of shRNAs are listed in Table S2 (Supporting Information).

### CCK‐8 Assay

Cells were seeded into 96‐well plates at a density of 1 × 10⁴ cells/well and assigned to different groups, each with six replicates. After removing the medium, 100 µL of fresh complete medium and 10 µL of CCK‐8 reagent (RM02823, ABclonal, China) were added per well. Plates were incubated at 37 °C in the dark for 1–2 h, and absorbance at 450 nm was recorded using Thermo Scientific Varioskan LUX Multi‐Mode Microplate Reader (RRID:SCR_02 6792).

### Immunoprecipitation (IP)

Microglial cells were transfected with the specified plasmid combinations. After 24 h, cells were lysed using IP cell lysis buffer, followed by centrifugation at 12500 × g for 20 min at 4 °C. The supernatant was incubated overnight at 4 °C with magnetic beads conjugated to anti‐Flag antibody (HY‐K0207, MedChemExpress, USA). The agarose beads were washed three times with cell lysis buffer, then boiled to elute bound proteins. The samples were subsequently subjected to SDS‐PAGE and western blotting analysis.

### Western Blotting Analysis

Protein expression levels were assessed by Western blotting as previously described.^[^ ^59^ ^]^ Briefly, 20 µg of total protein lysate was subjected to SDS‐PAGE and subsequently transferred onto polyvinylidene difluoride (PVDF) membranes. After blocking with 5% non‐fat milk for 1 h at room temperature, membranes were incubated overnight at 4 °C with primary antibodies. The next day, membranes were incubated for 1 h at 37 °C with the corresponding horseradish peroxidase (HRP)‐conjugated secondary antibodies. Following thorough washes with TBST, protein bands were visualized using an enhanced chemiluminescence (ECL) kit (HY‐K2005, MedChemExpress, USA). Each experiment was independently performed three times, and band intensities were quantified using ImageJ (RRID:SCR_0 03070) software. Detailed information on all antibodies used is provided in Table S3 (Supporting Information).

### Quantitative Real‐Time PCR (qRT‐PCR)

Total RNA was isolated from cells or tissue samples using TRIzol reagent (P5870328, Invitrogen, USA) following the manufacturer's instructions. Complementary DNA (cDNA) was synthesized using RT Master Mix according to the provided protocol. Quantitative PCR (qPCR) was performed with 2× SYBR Green qPCR Mix (AG11733, ACCURATE BIOLOGY, Changsha, China). Primer sequences used in this study are detailed in Table S4 (Supporting Information).

### CETSA

Control and drug‐treated cells (2 × 10^7^ per group) were cultured in 10‐cm dishes. After treatment, cells were washed with PBS, trypsinized, and pelleted by centrifugation at 300 × g for 4 min at room temperature. Pellets were washed twice with PBS and resuspended in 600 µL ice‐cold PBS. The suspension was divided into six aliquots of 100 µL each in PCR tubes. One tube was kept at room temperature, while the others were heated for 3 min at 37 °C, 42 °C, 47 °C, 52 °C, 57 °C, or 62 °C in the Bio‐Rad C‐1000 Thermal Cycler (RRID:SCR_01 9688). All samples underwent three freeze‐thaw cycles (liquid nitrogen for 1 minute and thaw at room temperature). After centrifugation at 12,000 rpm for 30 min at 4 °C, supernatants were collected for western blotting analysis.

### Molecular Docking

The crystal structure of LRP1 was retrieved from the Protein Data Bank (). Alp's 2D structure was drawn in ChemDraw 20.0 (RRID:SCR_01 6768) and converted into a 3D model, which was then optimized using Chem3D 20.0. Both structures, saved in PDB format, were used for molecular docking with AutoDock Vina (RRID:SCR_01 1958). The best binding pose was visualized and analyzed using PyMOL (v2.3.0) (RRID:SCR_000305) and Discovery Studio (v21.1.0) (RRID:SCR_0 08398).

### MST Assay

Recombinant Human LRP1 protein (20‐189aa; P09953, Solarbio) was labeled with red fluorescence using an Alexa Fluor® 647 Microscale Protein Labeling Kit (A20006, Thermo Fisher Scientific, USA). For the binding assay, a constant concentration of Alexa Fluor® 647‐labeled LRP1 was incubated with a series of concentrations of Alp. MST analysis was performed at 24 °C using standard capillaries on a Monolith NT.115 instrument (NanoTemper Monolith NT.115 (RRID:SCR_02 7500)). Binding affinity was determined by analyzing the thermophoretic traces using NanoTemper's proprietary analysis software.

### Transwell Assay

Cells in logarithmic growth phase and control cells were obtained by trypsinization and centrifugation. The cells were resuspended in serum‐free medium, and 1 × 10^5^cells were seeded into the upper chamber of Transwell inserts with an 8 µm pore size. The lower chamber was filled with 700 µL of medium containing 20% serum. After incubation at 37 °C for 24–48 h, cells were fixed with 4% paraformaldehyde and stained with 0.1% crystal violet. Migrated and invaded cells were then observed and quantified under Leica Microsystems (RRID:SCR_0 08960).

### EDU Assay

Cell proliferation was evaluated using an EDU assay kit (C0071, Beyotime, China) according to the manufacturer's instructions. Briefly, 1 × 10^5^ cells were seeded in 12‐well plates and cultured for 24 h. Cells were then incubated with 10 µm EDU (500 µL/well) for 2 h, followed by three PBS washes. After fixation with 4% paraformaldehyde and permeabilization with 0.3% triton X‐100 (15 min each), cells were treated with click‐reaction reagent for 30 min in the dark. Nuclei were stained with hoechst 33342, and fluorescence images from nine random fields per group were acquired using the Leica Microsystems (RRID:SCR_008960).

### BODIPY Staining

LD accumulation in microglia was assessed by BODIPY staining (HY‐D1614, MedChemExpress, USA) following established protocols.^[^ ^60^ ^]^ Microglial cells (4 × 10^4^ per coverslip) were cultured on glass coverslips for 12 h, then fixed with 4% paraformaldehyde for 30 min and rinsed with PBS. Cells were stained with BODIPY 493/503 and Hoechst 33 342 for 15 min at room temperature, washed twice with PBS, and imaged under a fluorescence microscope.

### ELISA

The conditioned medium from microglial cultures was collected and centrifuged at 4000 × g for 5 min at 4 °C to remove cellular debris. The resulting supernatant was aliquoted and stored at −80 °C until analysis. The concentrations of BDNF and IGF‐1 in the supernatant were quantified using commercial ELISA kits, strictly following the manufacturer's protocols.

### Co‐Culture of Microglia and RGCs

Microglia was treated according to the experimental groups and washed three times with PBS, followed by a 6 h incubation in fresh medium. The supernatant from the microglial culture was then collected and centrifuged at 4000 rpm for 10 min to remove cellular debris. The resulting MCM was added to the pre‐cultured primary RGCs system for a 24 h co‐culture period before subsequent analyses.

### Live/Dead Cell Imaging Assay

The viability of RGCs was assessed using a dual fluorescence staining method, which distinguishes cells based on differences in membrane integrity. The working solution consisted of 1 µm calcein‐AM and 0.5 µm ethidium homodimer‐1 dissolved in PBS buffer. The staining solution was added to cell samples after drug intervention, followed by a 30 min incubation in the dark. After staining, cells were rinsed with fresh PBS and observed under a fluorescence microscopy system. To ensure data reliability, images from six non‐overlapping fields were systematically captured for each experimental group under consistent microscopy parameters.

### Metabolic Assay

The concentrations of total cholesterol and triglycerides were measured using commercial assay kits (BC1985; BC0625; Solarbio, China), following the manufacturer's protocols. The absorbance at 500 and 420 nm was recorded to quantify cholesterol and triglyceride levels, respectively, using a Thermo Scientific Varioskan LUX Multi‐Mode Microplate Reader (RRID:SCR_026792). Each experiment was performed in triplicate to ensure accuracy and reproducibility.

### Flow Cytometry

Single‐cell suspensions were prepared from the retinal tissues of SD rats. BODIPY staining was performed according to the protocol provided with the staining kit. The samples were analyzed using the Thermo Fisher Attune Nxt Flow Cytometer (RRID:SCR_019590), and the data were processed and evaluated using FlowJo v10.8 (RRID:SCR_008520) software.

### Statistical Analysis

Data are presented in the form of individual data points and mean ± standard deviation, unless otherwise specified. Statistical analysis and chart production were both accomplished using GraphPad Prism 8 (RRID:SCR_002798). Statistical analysis between two independent groups was conducted using the Student *t*‐test, while among multiple groups, one‐way analysis of variance (ANOVA) combined with Bonferroni post hoc test or two‐way analysis of variance combined with Bonferroni post hoc test was used. The criterion for statistical significance was *p* <0.05.

## Conflict of Interest

The authors declare no conflict of interest.

## Author Contributions

M.W., Y.H., and J.Y. contributed to the work equally and should be regarded as co‐first authors. All authors listed made a significant contribution to the work reported and approved it for publication.

## Supporting information



Supporting Information

## Data Availability

The data that support the findings of this study are available on request from the corresponding author. The data are not publicly available due to privacy or ethical restrictions.
