# Supplementary material for: Alpinetin Nanoparticles Alleviate Optic Nerve Injury Induced by Acute Glaucoma via LRP1‐PPARγ Mediated Regulation of Microglial Lipid Metabolism
Source: Adv Sci (Weinh). 2025 Nov 12;13(5):e13270. doi: 10.1002/advs.202513270 (PMC12850317; doi:10.1002/advs.202513270)
Supplement: Supplementary file 1 — Supporting Information [file ADVS-13-e13270-s001.docx]

**Alpinetin Nanoparticles Alleviate Optic Nerve Injury Induced by Acute Glaucoma via LRP1-PPARγ Mediated Regulation of Microglial Lipid Metabolism**

**Miao Wei^1,2, a^, Yujia Huo^1,2, a^, Jingchang Yuan^1,2, a^, Xiao Fan^1,2^, Xiaochen Wang^1,2^, Sisi Tan^1,2^, Xi Gao^1,2^, Ruotong Ouyang^1,2^, Hong Li^1,2,^ ^*^**

**^1^ Department of Ophthalmology, The First Affiliated Hospital of Chongqing Medical University, Chongqing, People’s Republic of China**

**^2^ Chongqing Key Laboratory for the Prevention and Treatment of Major Blinding Eye Diseases, Chongqing Eye Institute, Chongqing Branch of National Clinical Research Center for Ocular Diseases, Chongqing, People’s Republic of China**

**^a^These authors contributed to the work equally and should be regarded as co-first authors.**

**^*^Corresponding author. E-mail addresses: lihong@hospital.cqmu.edu.cn**

| 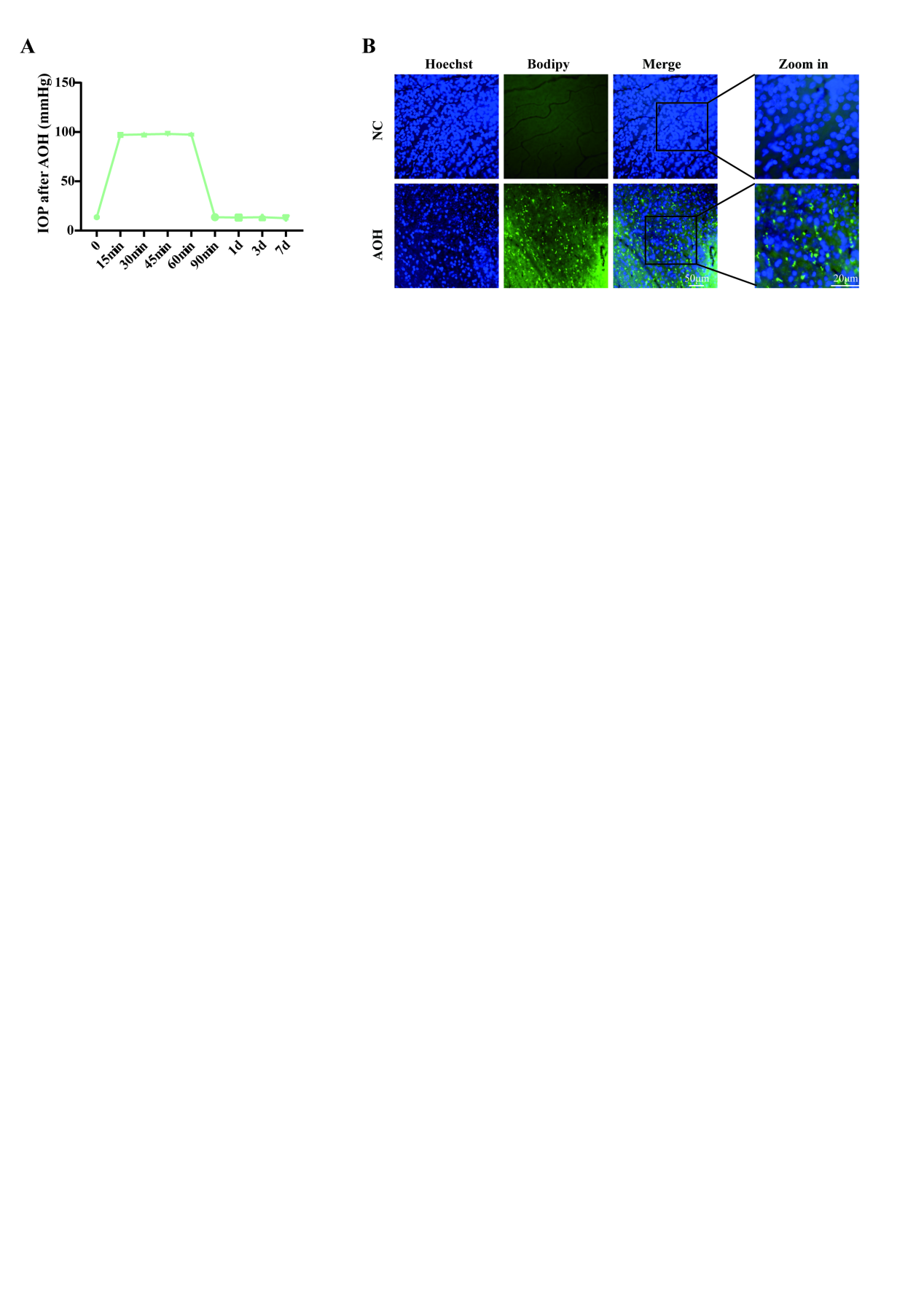 |
| --- |
| **Figure S1. Successful establishment of the AOH model and lipid dysregulation. (A)** IOP trajectories show a significant acute elevation in the AOH model group (mean ± SD, n=5). **(B)** Visualization of LD (BODIPY 493/503, green) in retinal flat mounts reveals pronounced accumulation in AOH retinas. Scale bar: 50 μm (n=4). |
| **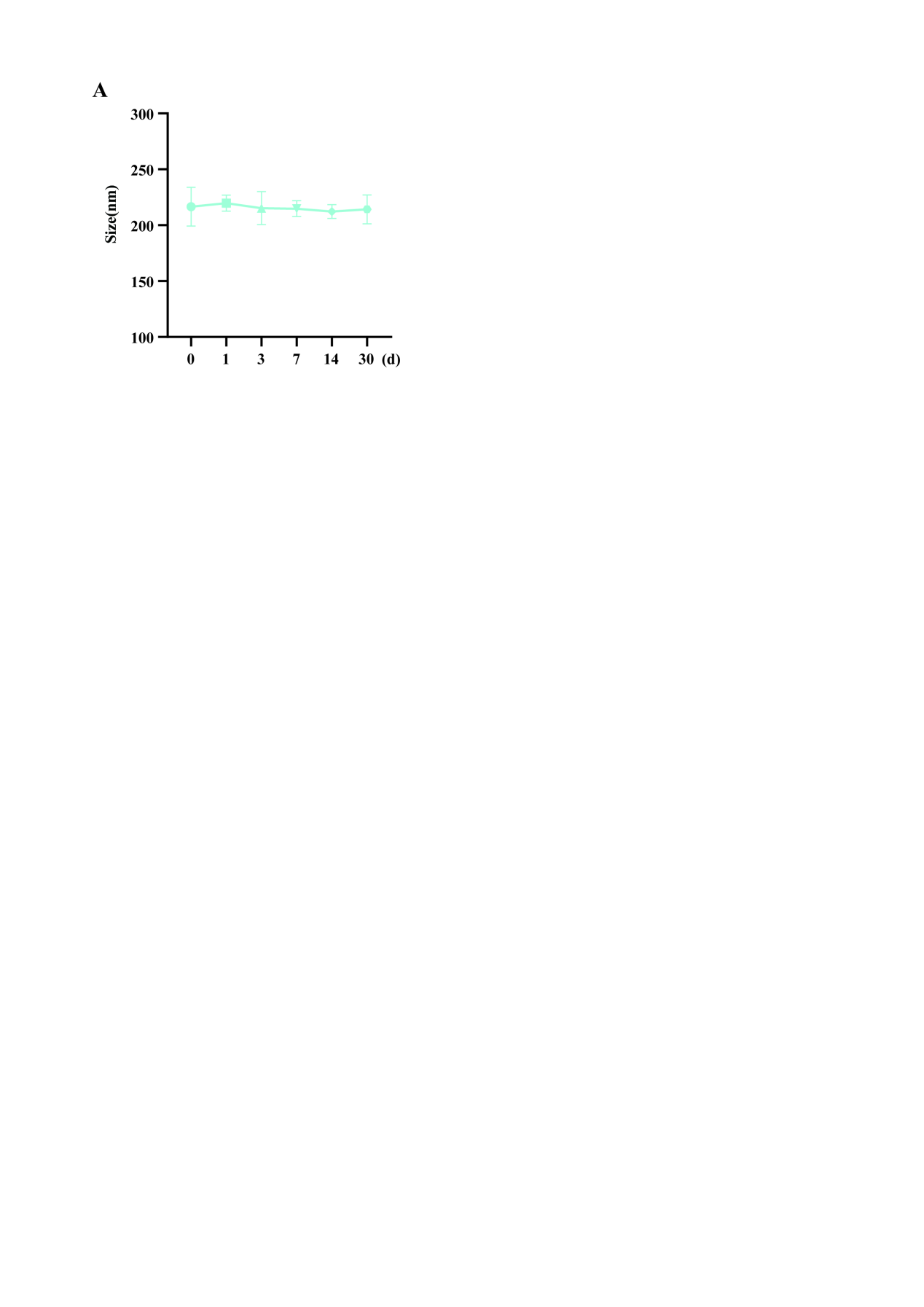** |
| **Figure S2. Assessment of AlpNPs stability. (A)The hydrodynamic size of AlpNPs was maintained over the tested duration, as measured by DLS, confirming their stability for subsequent applications (mean ± SD, n=3).** |

| 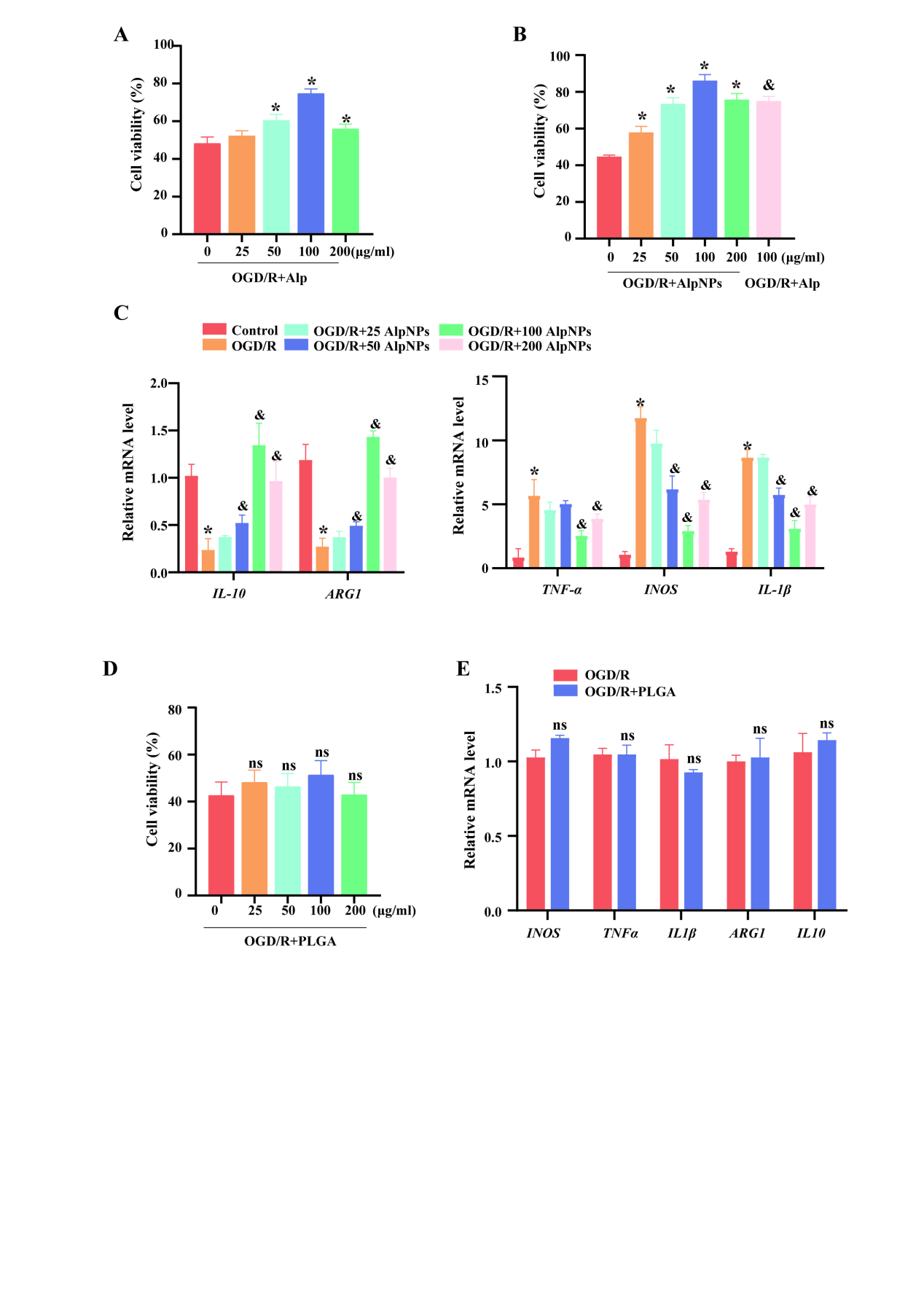 |
| --- |
| **Figure S3. Effects of Different Concentrations of AlpNPs and PLGA Carriers on Microglial Cells.** (A–B) CCK-8 assay assessing the viability of HMC3 cells under OGD/R conditions following treatment with Alp or AlpNPs. **p* < 0.05 vs. OGD/R group; &*p* < 0.05 vs. OGD/R + AlpNPs (100 μg/mL) group. (C) qPCR analysis of the expression of inflammation-related genes in ODG/ R-induced HMC3 cells after treatment with different concentrations of AlpNPs. **p* < 0.05 vs. Control group; &*p* < 0.05 vs. OGD/R group. (D) CCK-8 assay evaluating the effect of the PLGA carrier alone on HMC3 cell viability under OGD/R conditions, indicating no cytotoxicity. (E) qPCR analysis of inflammation-related genes expression in HMC3 cells after PLGA carrier treatment.Data are presented as mean ± SD. ns, not significant. |
| 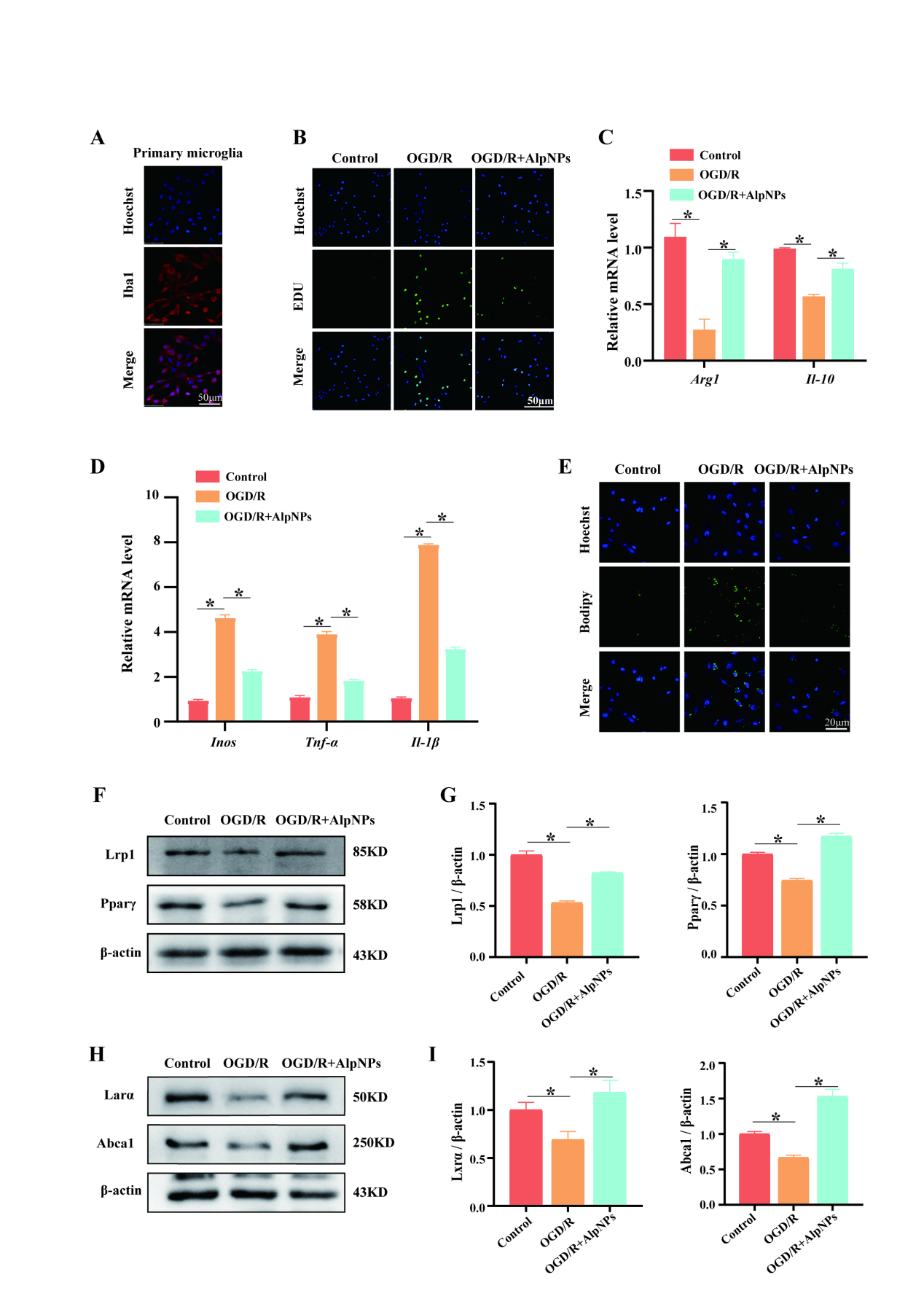 |
| **Figure S4. Effects of AlpNPs on Primary Microglia.** (A) Representative image of primary microglia isolated from neonatal SD rats labeled with Iba1. Scale bar: 50 μm. (B) EDU assay showing the proliferation of primary microglia under different treatments. Scale bar: 50 μm. (C–D) Bar graphs showing mRNA expression levels of inflammatory cytokines in primary microglia following various treatments. (E) Immunofluorescence analysis of LD accumulation in primary microglia. Scale bar: 50 μm. (F-I) Using β-actin as the loading control, western blotting was used to analyze the expressions of Lrp1, Pparγ, Lxrα and Abca1 in microglia after AlpNPs. (G) (I) The expression levels of (F) (H) proteins were quantitatively analyzed using ImageJ. The data represent the average ±SD of three independent experiments. **p* < 0.05. |
| **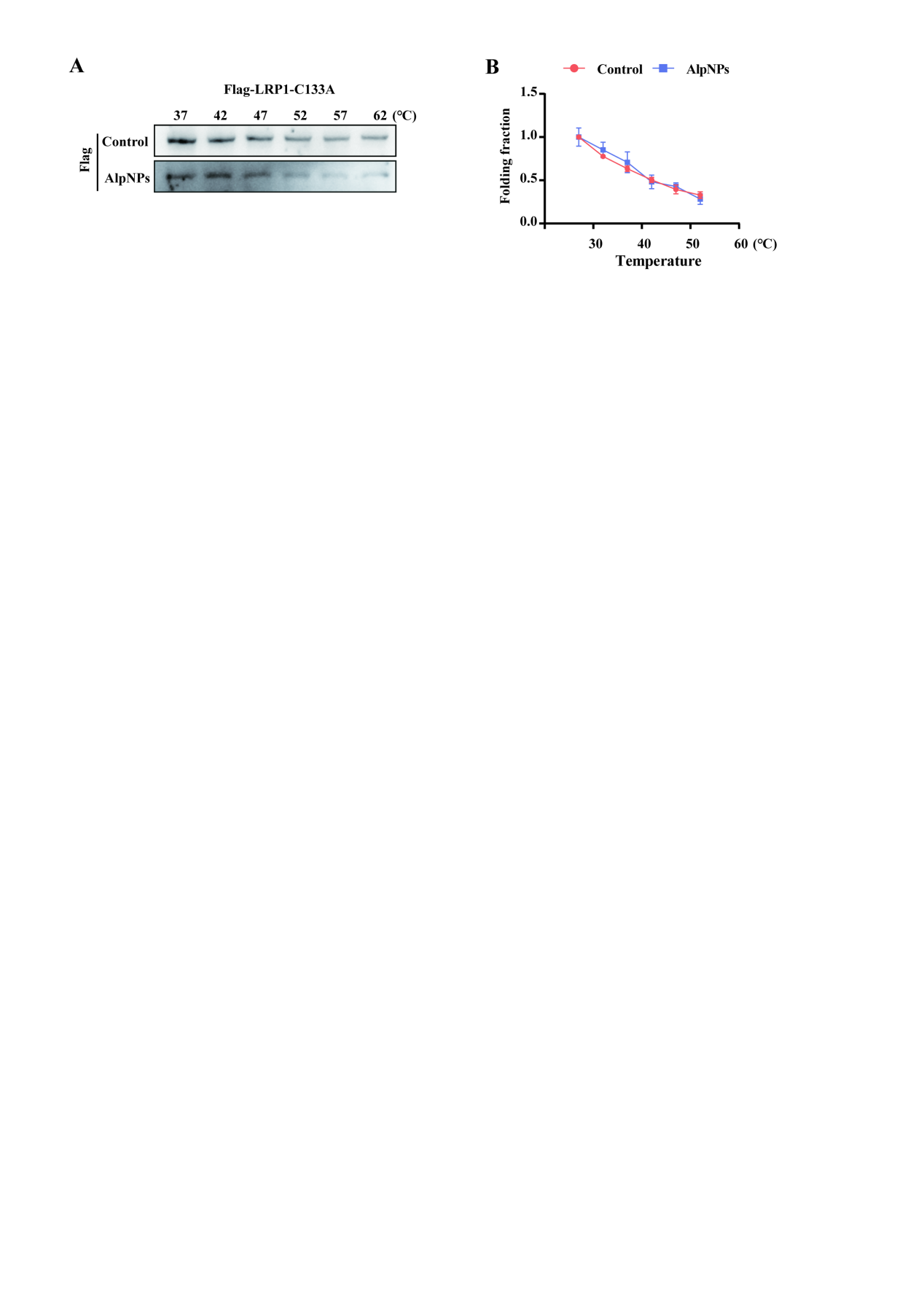** |
| Figure S5. The C133 site is crucial for the binding of Alpinetin to LRP1 (A-B) CETSA assay was used to analyze the effect of Alp on the thermal stability of the Flag-LRP1 C133A overexpressed in HMC3 cells. |
| 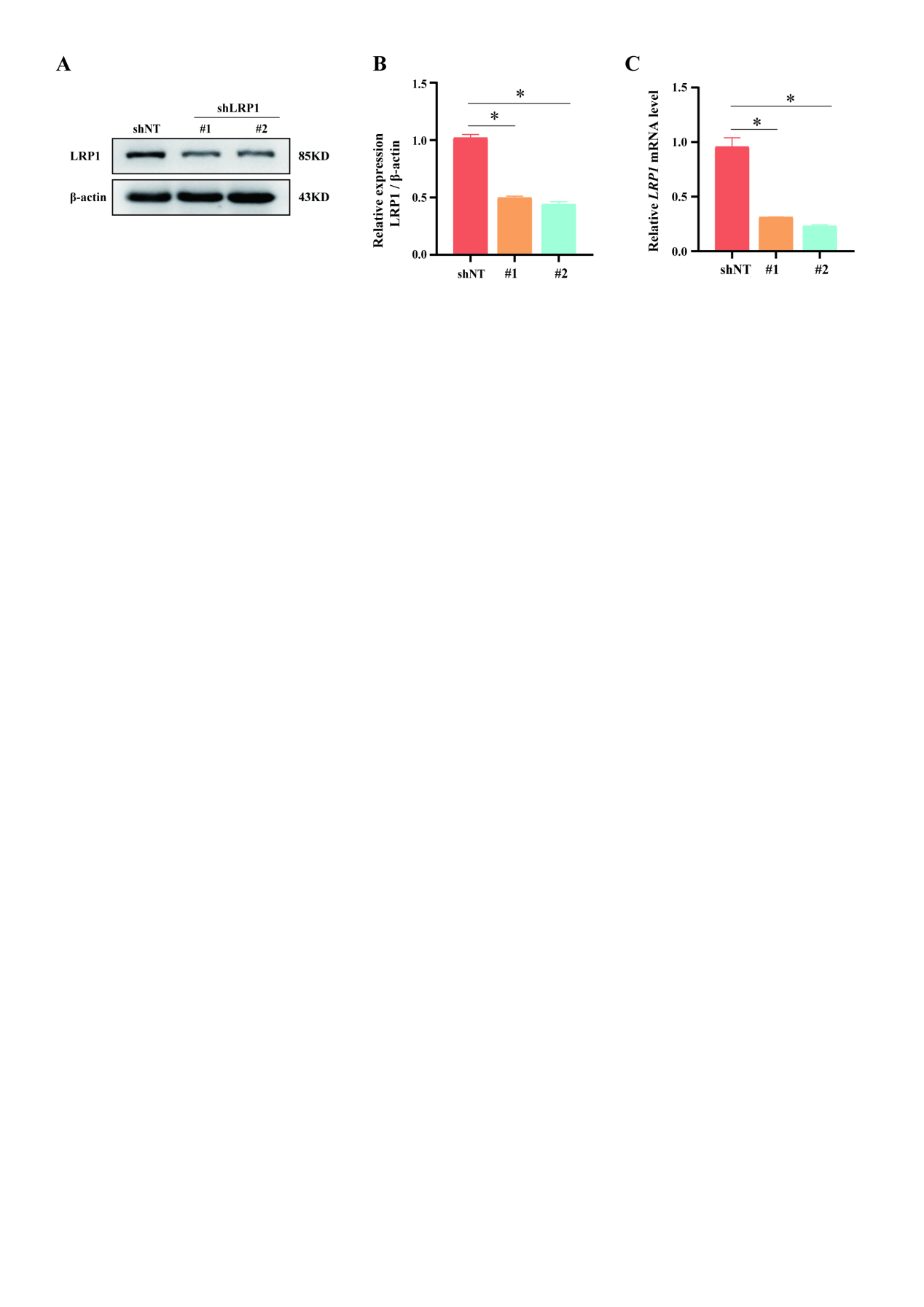 |
| **Figure S6. Validation of LRP1 Knockdown Efficiency in Microglia.** (A–B) Transfection with shLRP1 significantly reduced protein expression levels of LRP1 in HMC3 cells. (C) Transfection with shLRP1 significantly reduced mRNA levels of *LRP1* in HMC3 cells. Data are presented as mean ± SD. ****p* < 0.05** indicates a statistically significant difference. |
| 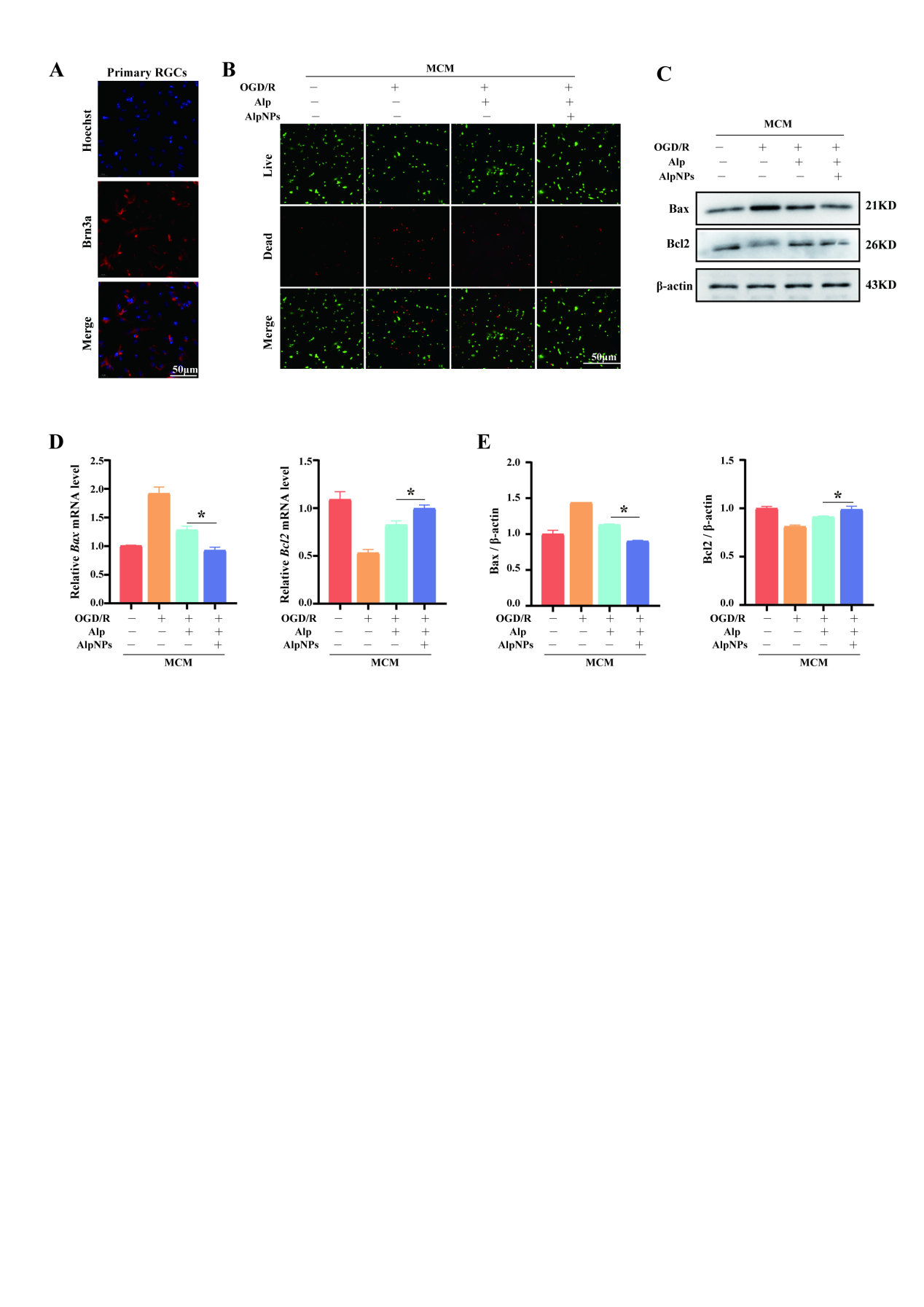 |
| **Figure S7. Effects of Alp or AlpNPs Treated Microglia on Primary RGCs Survival.** (A)Representative immunofluorescence image of primary RGCs isolated from Brn3a-labeled SD neonatal rat retina. Scale bar: 50 μm. (B) Representative images of live (green) and dead (red) RGCs co-cultured with conditioned media from microglia treated with Alp or AlpNPs. Scale bar: 50 μm. (C-E) Bar graphs showing protein and mRNA expression levels of Bax and Bcl2 under different treatment conditions. Data represents the mean ± SD of three independent experiments. **P* < 0.05. |
| 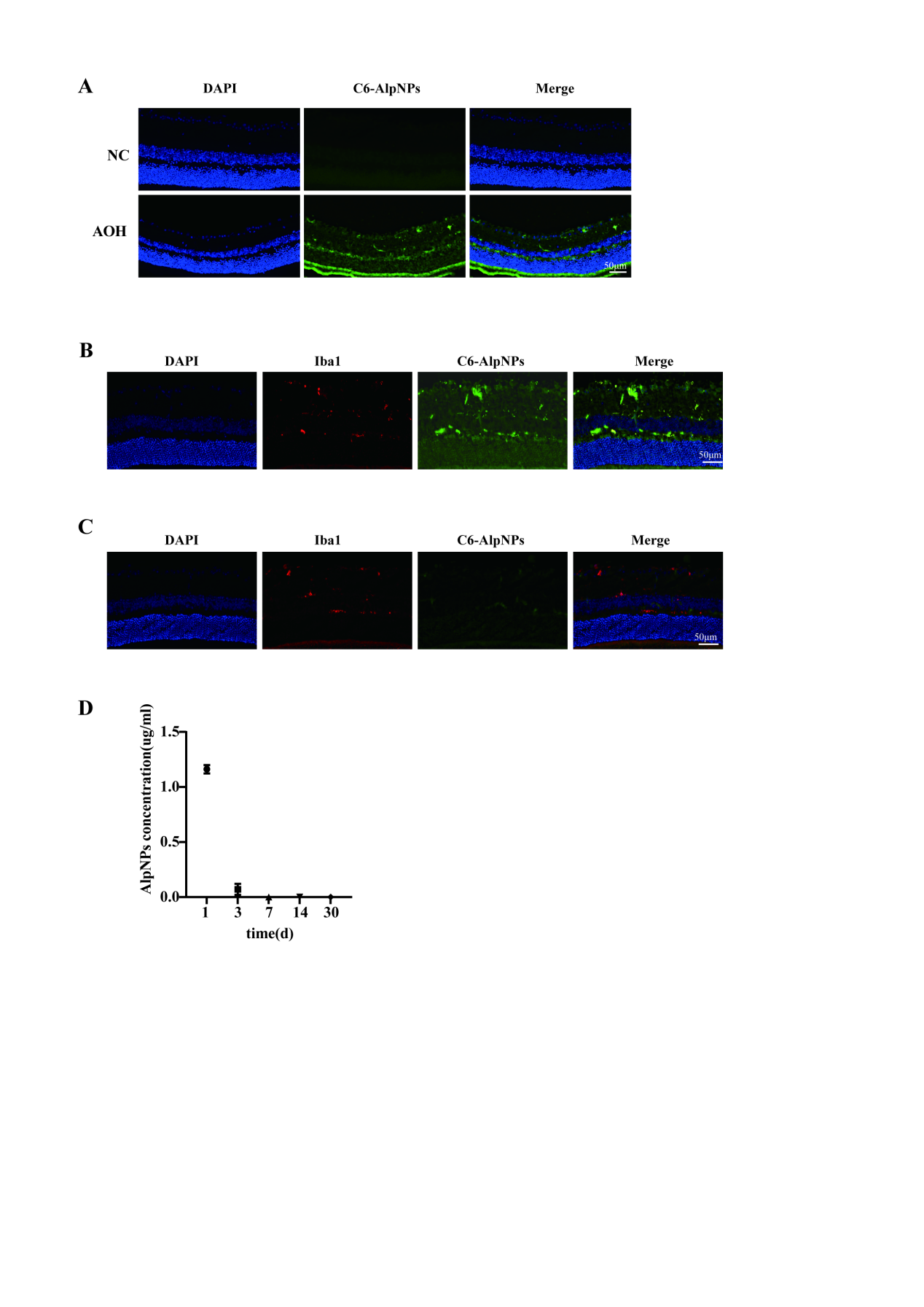 |
| **Figure S8. In Vivo Retinal Distribution of AlpNPs.** (A)Representative immunofluorescence images of retinal distribution in SD rats three days after intravitreal injection of C6-AlpNPs.Scale bar: 50 μm. (B)Representative immunofluorescence images of Iba1 in the retina of SD rats 3 days after intravitreal injection of C6-AlpNPs.Scale bar: 50 μm. (C)Representative immunofluorescence images of Iba1 in the retina 2 weeks post-intravitreal injection of C6-AlpNPs, showing no detectable green fluorescence. Scale bar: 50 μm. (D) Changes in the concentration of Alp in the retina after intravitreal injection of AlpNPs. |
| 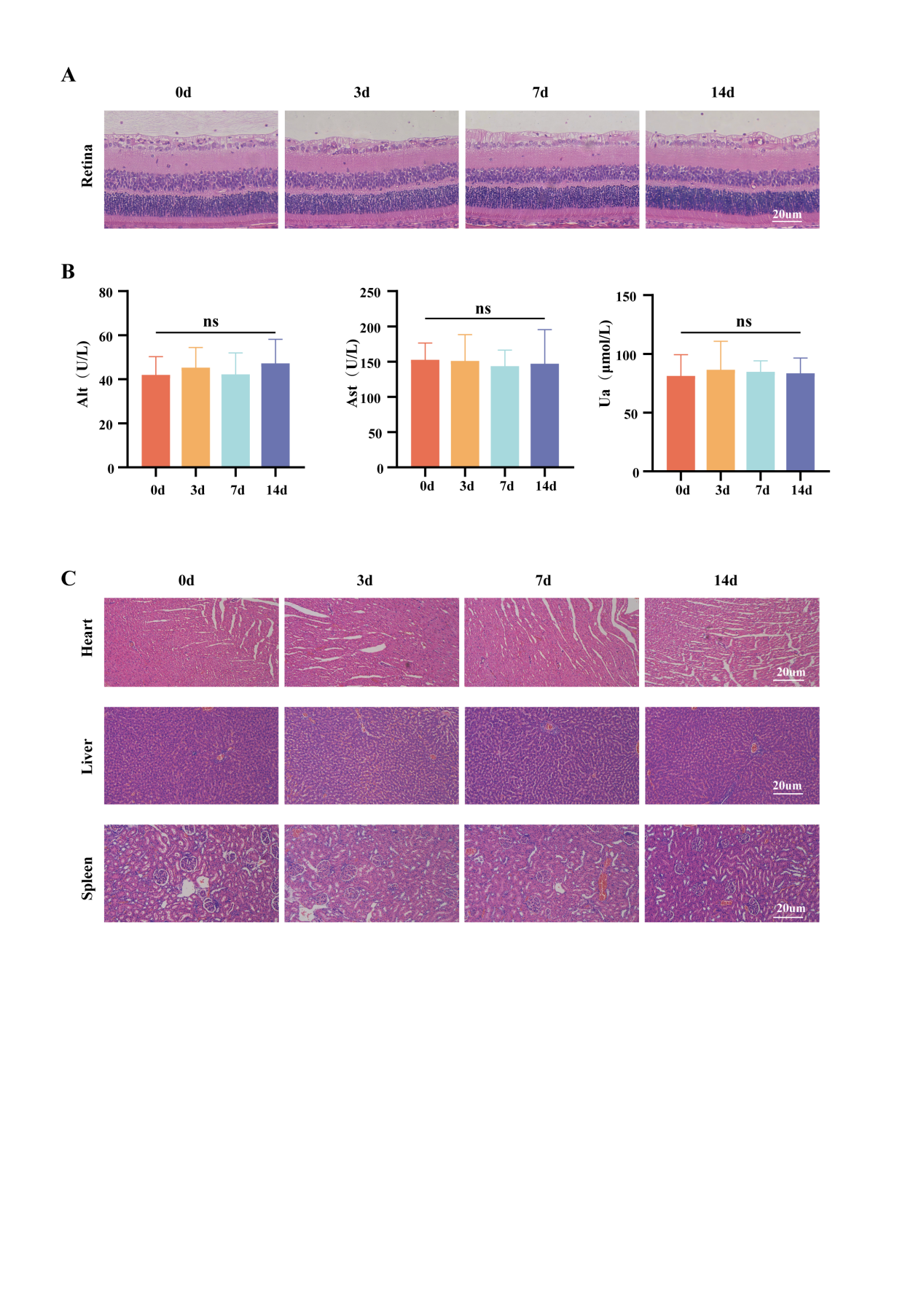 |
| **Figure S9. Biosafety evaluation of AlpNPs. (A) Retinal morphology (H&E staining) after AlpNPs injection. Scale bar: 20 μm (n=4). (B) Blood biochemistry at indicated time points showed no significant alterations (mean ± SD, n=4; ns, not significant). (C) Histological analysis of major organs reveals no obvious damage throughout the observation period. Scale bar: 20 μm (n=4).** |
| **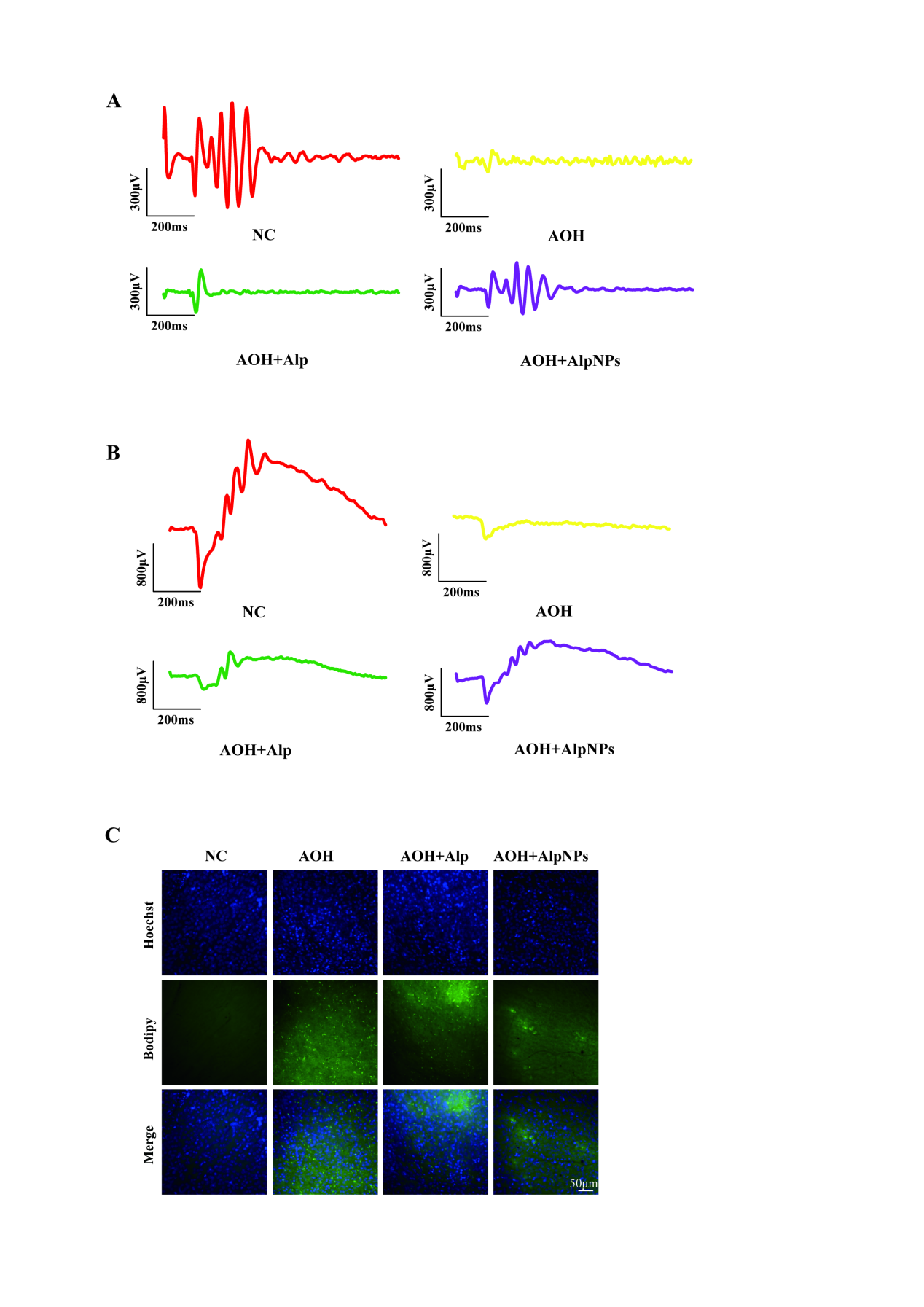** |
| **Figure S10. AlpNPs improves visual function and reduces lipid accumulation in the AOH model. (A-B) Representative images of** 3.0cd’s/m^2^ **and OPs in each group (n=4). (C) BODIPY 493/503 staining plots of retinal patches in each group (n=4), Scale bar: 50 μm.** |

Table S1 Clinical characteristies of patients

|  | ARC | POAG | PACG |
| --- | --- | --- | --- |
| Case | 16 | 17 | 19 |
| Age | 62(51-78) | 64(50-82) | 66(54-79) |
| Sex (Male/Female) | 8/8 | 10/7 | 8/11 |
| Diabetes | - | - | - |
| Hypertension | - | - | - |
| Hyperlipidemia | - | - | - |

Table S2 RNA oligo for LRP1-shRNA transfection

| Gene | Sense | Antisense |
| --- | --- | --- |
| shLRP1#1 | CAGGAGGATGTTTGCACACTT | AAGTGTGCAAACATCCTCCTG |
| shLRP1#2 | GATGAAGACTTCTGCCAGAAT | ATTCTGGCAGAAGTCTTCATC |

Table S3 Antibody information

| Target | Product information | Diluted concentration | RRID |
| --- | --- | --- | --- |
| Inos | Wanleibio；#WL0992 | WB: 1:1000 | AB_3675335 |
| Il-1β | Proteintech; #26048-1-AP | WB: 1:1000 | AB_2880351 |
| Tnf-α | Abmart；# PY19810 | WB: 1:1000 | AB_2920645 |
| Arg1 | Wanleibio; #WL02825 | WB: 1:500 | AB_3674058 |
| Il-10 | Abmart；#TD6894 | WB: 1:1000 | AB_2936848 |
| CD206 | abcam；#ab64693 | IF: 1:200 | AB_1523910 |
| CD86 | BD Biosciences; # 562432 | IF: 1:200 | AB_11153866 |
| Brn3a | Novus; # NB110-57427 | IF: 1:200 | AB_2167517 |
| Lrp1 | abcam；#ab92544 | WB: 1:1000 | AB_2234877 |
| Plin2 | Proteintech；#15294-1-AP | IF: 1:200 | AB_2878122 |
| PPARγ | Proteintech; #16643-1-AP | WB: 1:1000 | AB_10596794 |
| LXRα | Proteintech; #14351-1-AP | WB: 1:1000 | AB_10640525 |
| ABCA1 | CST; #96292 | WB: 1:1000 | AB_3661872 |
| Iba1 | ThermoFisher; # MA5-43700  Wako; #019-19741 | IF: 1:200 | AB_2912632  AB_839504 |
| Flag | Abmart；#M20008 | WB: 1:5000 | AB_2713960 |
| Bax | Abmart；#T40051 | WB: 1:1000 | AB_2910262 |
| Bcl2 | Abmart；#T40056 | WB: 1:1000 | AB_2929011 |
| β-actin | Proteintech;#66009-1-Ig | WB: 1:5000 | AB_2687938 |
| Anti-Rabbit HRP | Abmart; #M21002 | WB: 1:10000 | AB_2713951 |
| Anti-mouse HRP | Abmart; #M21001 | WB: 1:10000 | AB_2713950 |
| Alexa Fluor 594  Goat anti-mouse IgG | Proteintech; #RGAM004 | IF: 1:400 | AB_3073502 |
| Alexa Fluor 488  Goat anti-rabbit IgG | Proteintech; #RGAR002 | IF: 1:400 | AB_3073506 |

Table S4 Primers for quantitative real-time PCR.

| Gene | Forward | Reverse |
| --- | --- | --- |
| *R-Tnf-α* | CACACGAGACGCTGAAGTAGT | GTGAGTTCCGAAAGCCCATTG |
| *R-Il-1β* | CAAAAATGCCTCGTGCTGTCT | TCGTTGCTTGTCTCTCCTTGT |
| *R-Inos* | CTTGGAGCGAGTTGTGGATTG | CCTCTTGTCTTTGACCCAGTAGC |
| 1. *Il-10* | CAGACCCACATGCTCCGAGA | CAAGGCTTGGCAACCCAAGTA |
| *R-Arg1* | CTGAAGTGGACAAGCTGGGA | CGTAGCCGGGGTGAATACTG |
| *R-Bax* | AAGACAGGGGCCTTTTTGCT | TGTTGTCCAGTTCATCGCCA |
| *R-Bcl2* | GCCTGAGAGCAACCGGAC | GTAGCGACGAGAGAAGTCATCC |
| *R- Cleaved caspase-3* | CAACAACGAAACCTCCGTGG | ACACAAGCCCATTTCAGGGT |
| *R-β-actin* | GAGGGTTACGCGCTCCC | AATGTCACGCACGATTTCCCT |
| *H-IL-1β* | TGAAGCAGCCATGGCAGAAG | GGTCGGAGATTCGTAGCTGGA |
| *H-TNF-α* | TCCTCTCTGCCATCAAGAGC | ATCCCAAAGTAGACCTGCCC |
| *H-INOS* | CTTGGAGCGAGTTGTGGATTG | CCTCTTGTCTTTGACCCAGTAGC |
| *H-ARG1* | TCATCTGGGTGGATGCTCACAC | GAGAATCCTGGCACATCGGGAA |
| *H-IL-10* | TCTCCGAGATGCCTTCAGCAGA | TCAGACAAGGCTTGGCAACCCA |
| *H-LRP1* | CAACGGCATCTCAGTGGACTAC | TGTTGCTGGACAGAACCACCTC |
| *H-β-actin* | CACCATTGGCAATGAGCGGTTC | AGGTCTTTGCGGATGTCCACGT |
